# Inactivation of Genes for Antigenic Variation in the Relapsing Fever Spirochete *Borrelia hermsii* Reduces Infectivity in Mice and Transmission by Ticks

**DOI:** 10.1371/journal.ppat.1004056

**Published:** 2014-04-03

**Authors:** Sandra J. Raffel, James M. Battisti, Robert J. Fischer, Tom G. Schwan

**Affiliations:** 1 Laboratory of Zoonotic Pathogens, Rocky Mountain Laboratories, National Institute of Allergy and Infectious Disease, National Institutes of Health, Hamilton, Montana, United States of America; 2 Division of Biological Sciences, The University of Montana, Missoula, Montana, United States of America; Medical College of Wisconsin, United States of America

## Abstract

*Borrelia hermsii*, a causative agent of relapsing fever of humans in western North America, is maintained in enzootic cycles that include small mammals and the tick vector *Ornithodoros hermsi*. In mammals, the spirochetes repeatedly evade the host’s acquired immune response by undergoing antigenic variation of the variable major proteins (Vmps) produced on their outer surface. This mechanism prolongs spirochete circulation in blood, which increases the potential for acquisition by fast-feeding ticks and therefore perpetuation of the spirochete in nature. Antigenic variation also underlies the relapsing disease observed when humans are infected. However, most spirochetes switch off the bloodstream Vmp and produce a different outer surface protein, the variable tick protein (Vtp), during persistent infection in the tick salivary glands. Thus the production of Vmps in mammalian blood versus Vtp in ticks is a dominant feature of the spirochete’s alternating life cycle. We constructed two mutants, one which was unable to produce a Vmp and the other was unable to produce Vtp. The mutant lacking a Vmp constitutively produced Vtp, was attenuated in mice, produced lower cell densities in blood, and was unable to relapse in animals after its initial spirochetemia. This mutant also colonized ticks and was infectious by tick-bite, but remained attenuated compared to wild-type and reconstituted spirochetes. The mutant lacking Vtp also colonized ticks but produced neither Vtp nor a Vmp in tick salivary glands, which rendered the spirochete noninfectious by tick bite. Thus the ability of *B. hermsii* to produce Vmps prolonged its survival in blood, while the synthesis of Vtp was essential for mammalian infection by the bite of its tick vector.

## Author Summary

*Borrelia hermsii*, an agent of tick-borne relapsing fever when infecting humans, employs antigenic variation of the variable major proteins (Vmps) to escape the host immune response. This mechanism allows the bacteria to persist in the blood of a mammal, which increases their potential for acquisition by their tick vector *Ornithodoros hermsi*. Once in the tick, the bacteria move from the midgut to salivary glands where the Vmps are replaced with another major surface protein, the variable tick protein (Vtp). We constructed two mutants, one that was unable to produce a Vmp (Vmp^−^) and another that was unable to produce Vtp (Δ*vtp*). The Vmp^−^ mutant could not reach as high bacterial levels in the blood of mice when infected by needle-inoculation and tick bite compared to the parent strain, and was incapable of relapsing. The Δ*vtp* mutant was able to colonize ticks, but was non-infectious by tick bite. Our study provides insight into the roles of the Vmps and Vtp in the infectivity of *B. hermsii* by showing the importance of antigenic variation for prolonging bacteria levels in the host as well as the requirement of Vtp for mammalian infection by the bite of its tick vector.

## Introduction

*Borrelia hermsii* is one of many human pathogens that escapes the host’s adaptive immune response by changing its outer surface proteins through antigenic variation [1]–[3]. This mechanism of immune evasion involves a large repertoire of genes that encode dominant outer surface proteins, the variable major proteins (Vmps), only one of which is produced by a single spirochete at any one time [4]. Most *B. hermsii* cells in a bloodstream population produce the same Vmp, which defines the serotype that is antigenically distinct from other populations of cells that precede and follow it during the course of infection. However, within the population there are rare antigenic variants that arise spontaneously, which have different Vmps and can be the founders for the next dominant serotype. This switch in the spirochete that results in the production of a different Vmp occurs by gene conversion, a nonreciprocal gene transfer of a silent promoter-less *vmp* gene cassette, which is recombined into the single *vmp* expression site near the telomere of the linear plasmid lp28-1 [1], [5]–[7].

As the spirochetes achieve high cell densities in the blood, the bacteria are cleared by the host’s immune response, dominated by an IgM antibody response directed at the Vmp on the surface of the spirochetes [8]–[12]. Spirochetes coated by a Vmp that is antigenically distinct from the majority of the population avoid this immune attack, replicate and produce a new population of bacteria in the host (the relapse), which in turn is attacked by a new IgM antibody response. This process of antigenic variation can repeat for many cycles in the mammalian host [4], [13], [14]. When people are infected with this spirochete, the resulting illness is characterized by repeated cycles of acute febrile episodes and remission when the patient feels almost normal, hence the name relapsing fever. In nature, this spirochete’s ability to produce repeated bacteremias in the peripheral blood of small mammals increases its potential to be acquired by its obligate, fast-feeding tick vector *Ornithodoros hermsi* [15].

Each antigenically distinct *B. hermsii* Vmp is encoded by a different *vmp* cassette. To date, 59 silent cassettes have been identified [16] and the Vmps they encode segregate into two families based on their molecular mass. The variable small proteins (Vsps) are approximately 22 kDa, while the variable large proteins (Vlps) are approximately 37 kDa [17], [18]. The silent cassettes are located on the lp28-1 *vmp* expression plasmid and other linear plasmids of similar size [16].

**Figure 1:**
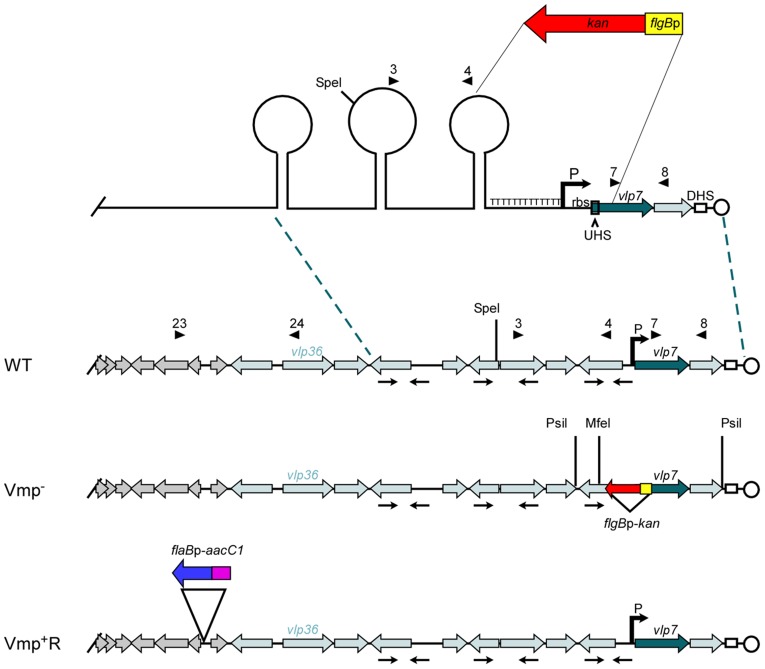
Diagram of the lp28-1 plasmids for the wild-type, Vmp^−^ mutant and reconstituted Vmp^+^R strains. Schematic of a portion of the *B. hermsii* linear plasmid lp28-1 contained in the wild-type (WT), Vmp-minus mutant (Vmp^−^), and reconstituted (Vmp^+^R) strains. A region of the lp28-1 plasmid including the 5′ end of *vlp7*, Upstream Homology Sequence (UHS), promoter (P) and ribosome binding site (rbs), T-rich region, and proximal inverted repeat was replaced with the *flgB*p-*kan* cassette to construct the Vmp^−^ strain. DHS = Downstream Homology Sequence. Arrowheads with numbers indicate primer number and location. Arrows oriented toward each other indicate inverted repeats that coincide with the stems in the potential stem loop structures. ORFs in grey are plasmid replication genes. Light-shaded ORFs indicate *vmp* silent cassettes. Dark-shaded ORF is the *vmp* in the expression site (*vlp7*). Restriction sites for Southern blot analysis are indicated on the Vmp^−^ plasmid. The reconstituted strain Vmp^+^R has the wild-type lp28-1 containing the *flaB*p-*aacC1* cassette.

The mechanism of antigenic variation in *B. hermsii* has been studied extensively by examining the DNA sequences surrounding the *vmp* gene in the active expression site and comparing them to the DNA surrounding the gene in its silent location for several different serotypes [6], [16], [17], [19]–[21]. Previously published sequences surrounding the expression locus and 12 kb of DNA immediately upstream on the lp28-1 plasmid [Genbank accession numbers DQ218042 (*B. hermsii* HS1) and CP000273 (*B. hermsii* DAH)] [16], [19] indicated key genetic elements that are involved in both the DNA recombination and expression of a *vmp*. A sigma-70 type promoter and a ribosome-binding site (Fig. 1) allow for production of the Vmp. Directly upstream of the promoter is a string of 13 T residues (16 T residues in the HS1 strain) [17], [19], [20] that enhances transcription from the promoter [22]. Upstream of this T-rich region are three sets of imperfect inverted repeats that potentially form stem-loop structures. These inverted repeats are unique to the expression plasmid lp28-1 and therefore may play a role in expression or recombination of the *vmp* gene [19]. The Upstream Homology Sequence (UHS) and the Downstream Homology Sequence (DHS) flanking the *vmp* may be necessary for the recombination of a new *vmp* gene into the expression site [16], [17]. The UHS contains approximately 60 nucleotides and encompasses the transcriptional start site and part of the coding sequence of the signal peptide of the *vmp*, and the DHS is a 214 bp nucleotide sequence downstream of the coding sequence.

When *O. hermsi* ticks acquire the spirochetes from an infected mammal, the bacteria switch from producing the bloodstream Vmp to producing another major outer surface protein, the variable tick protein (Vtp) [23], [24]. The *vtp* gene is transcribed from its own promoter, which is located on a larger linear plasmid that varies in size from 35 to 53 kb among different *B. hermsii* isolates [25]–[27]. In contrast to the repertoire of *vmp* cassettes, there is only one copy of *vtp* in the spirochete’s genome [26]. When *B. hermsii* expresses a *vmp* gene, the *vtp* expression is down-regulated [23], [26]. However, when *B. hermsii* is acquired by ticks, this reciprocal synthesis involving the two expression sites is reversed, with Vtp replacing the bloodstream Vmp [26]. Thus two key components of the *B. hermsii* life cycle include the ability to sequentially produce antigenically distinct serotypes in the mammalian bloodstream, and to replace the Vmps with a different major surface protein, Vtp, when infecting ticks.

We report here the first genetic study of the roles of the Vmps and Vtp in the infectivity and transmissibility of *B. hermsii* in mice and ticks. We constructed a mutant (Vmp^−^) that was unable to produce a Vmp or undergo antigenic variation. We then reconstituted the mutation to wild-type (Vmp^+^R), and followed the strains through the infectious cycle with ticks and mice. We show that the Vmp^−^ mutant colonized ticks but caused a reduced initial spirochetemia in immunocompetent mice and was unable to relapse, compared to the wild-type and Vmp^+^R reconstituted strains. We also show that the Vmp^−^ mutant maintained a persistent infection in immunodeficient SCID mice, but again produced lower cell densities compared to the wild-type and Vmp^+^R strains. We also tested a mutant (Δ*vtp*) unable to produce Vtp and show that, while this mutant was also able to persistently colonize ticks, the inability of *B. hermsii* to produce Vtp rendered the spirochetes noninfectious by tick bite.

## Results

### Construction of a Vmp-minus mutant (Vmp^−^)

We constructed a Vmp-minus mutant (Vmp^−^) by replacing a segment of DNA on plasmid lp28-1 involved in the expression and recombination of a new *vmp* gene with a kanamycin-resistance cassette (Fig. 1). The deleted region included the first 340 bp of the 1107 bp *vlp7* gene in the expression site, the UHS, the promoter and ribosome binding site, the T-rich region, and the proximal inverted repeat (Fig. 1). PCR analysis of the mutant confirmed that the kanamycin-resistance cassette was inserted into the lp28-1 in place of the deleted region (data not shown). Sequencing of the amplicons also confirmed the insertion.

Southern blot analysis of genomic DNA from the wild-type spirochete and two Vmp^−^ clones digested with *Psi*I or *Mfe*I (sites indicated on Fig. 1) and probed with *vlp7* or *kan* probes also confirmed that the mutation occurred in the telomeric expression site and not in the silent *vlp7* cassette on another plasmid (Genbank accession no. CP000274) [16]) or into a rare long expression plasmid in which the expression locus is not at the telomere but more internal on the plasmid [7] (Fig. S1).

### Construction of the reconstituted strain (Vmp^+^R)

Since the location near the telomere of lp28-1 may be important for efficient switching of a new *vmp* gene into the expression site, we did not believe the Vmp^−^ mutant could be complemented in trans with the *B. hermsii* shuttle vector pBhSV2 [28]. Therefore, a reconstituted strain was constructed by replacing the mutant expression plasmid with a wild-type lp28-1 marked with a gentamicin-resistance cassette (*flaB*p-*aacC1*)(Fig. 1), using a method of plasmid incompatibility applied previously to *B. burgdorferi* [29]–[34]. The reconstituted strain was gentamicin-resistant and kanamycin-sensitive, and PCR analysis of the expression locus indicated that an intact locus with a full-length *vlp7* in the expression site was present in the Vmp^+^R strain (data not shown).

**Figure 2:**
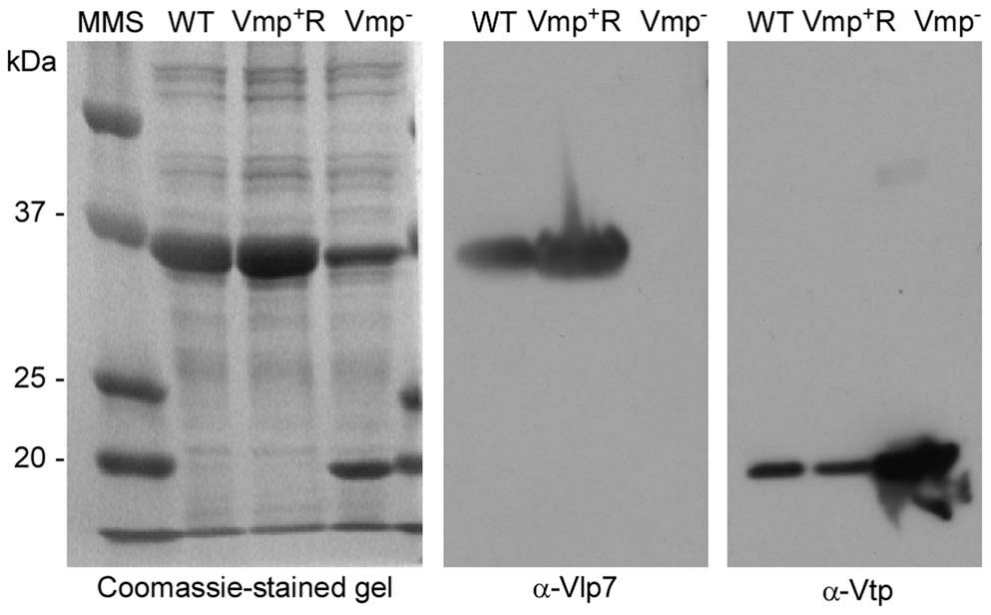
The Vmp^−^ mutant *B. hermsii* produces Vtp but not Vlp7. Coomassie-stained PAGE gel of whole-cell lysates of the wild-type (WT), Vmp^−^ mutant, and reconstituted (Vmp^+^R) strains (left panel) and immunoblots probed with anti-Vlp7 (middle panel) and anti-Vtp (right panel) monoclonal antibodies show that the wild-type and reconstituted strains produced Vlp7 and the mutant produced Vtp. Molecular mass standards are shown at left in kDa.

Genomic DNA of the wild-type, Vmp^−^, and Vmp^+^R strains was separated in a reverse-pulse-field gel, Southern blotted and probed for *vlp7*, *vlp36* (a silent cassette located on lp28-1) (Fig.1), *kan*, and *aacC1* (Fig. S2). The *vlp7* probe hybridized to two plasmids in all strains; one corresponding to the linear plasmid containing the silent *vlp7* cassette and the other to the expression plasmid lp28-1 containing *vlp7* in the expression site. The *vlp36* probe hybridized only to lp28-1 in all 3 strains. The *kan* probe hybridized only to the lp28-1 plasmid in the mutant, and the *aacC1* probe hybridized only to the reconstituted strain lp28-1. Immunoblot analysis confirmed that the wild-type and Vmp^+^R strains produced Vlp7, whereas Vmp^−^ did not and produced Vtp instead (Fig. 2).

### Vmp^−^ mutant does not cause a relapse in mice infected by needle inoculation

**Figure 3:**
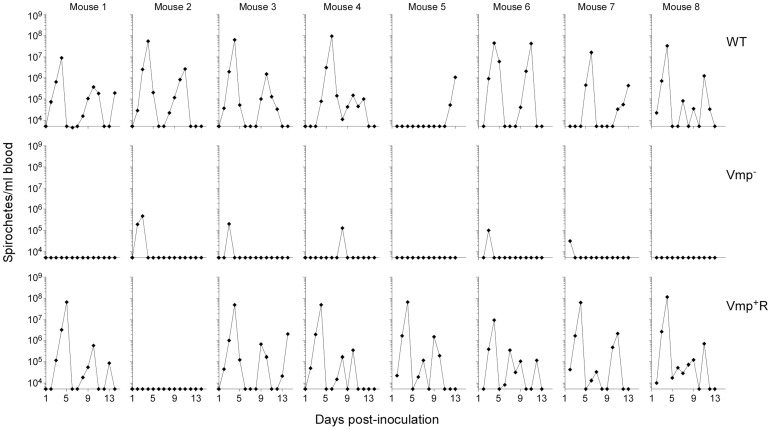
The *vmp* expression site is required for *B. hermsii* to relapse in mice following needle-inoculation. RML mice were injected ip with 500 spirochetes of the wild-type (WT), Vmp^−^ mutant (Vmp^−^), or reconstituted (Vmp^+^R) strains. Mice 1–4 were sampled on days 1–14 and mice 5–8 sampled on days 2–14 post-infection and the numbers of spirochetes per ml of blood were determined by QPCR. Each plot represents the data from an individual mouse. The lack of a detectable relapse spirochetemia in the mice infected with the Vmp^−^ mutant compared to the WT and Vmp+R strains was statistically significant (Fisher’s Exact Test, *P* = 0.0001).

Since the Vmp^−^ mutant was unable to produce any Vmp, we determined whether these spirochetes could persist in mice. Groups of eight RML mice were inoculated with 500 wild-type, Vmp^−^ or Vmp^+^R spirochetes and bacterial densities in the blood were assessed for 14 days by Quantitative PCR (QPCR) (Fig. 3). All mice inoculated with the wild-type spirochetes became infected and relapsed. While an initial spirochetemia was detected in 5 out of 8 mice infected with Vmp^−^, none of them relapsed. Among the 8 mice inoculated with Vmp^+^R, 7 of the animals relapsed, while one animal (mouse 2) had no detectable spirochetemia by QPCR.

**Figure 4:**
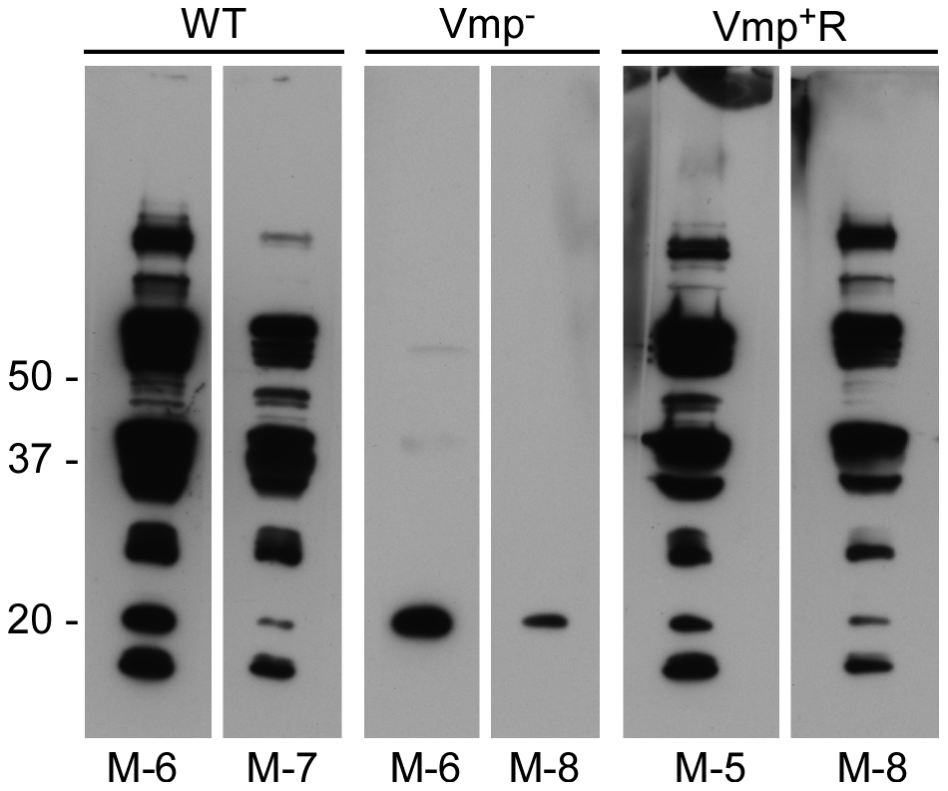
Mice inoculated with the Vmp^−^ mutant *B. hermsii* have a reduced serological response to infection. Whole-cell lysates of wild-type spirochetes were separated by PAGE, transferred to nitrocellulose membranes and analyzed with mouse serum (diluted 1:500) collected at 4 weeks post infection. Two mice with representative reactivity from each group (WT, Vmp^−^ or Vmp^+^R) of eight mice infected by needle inoculation are shown. Molecular mass standards are shown at left. The individual mouse number is indicated on the bottom of each panel.

Serum samples were collected from all 24 animals at 4 weeks post-infection, to compare the degree of antibody response the mice produced as a result of their infection with either Vmp^−^, wild-type or Vmp^+^R. The serum samples from mice that relapsed during their infection (i.e. those infected with wild-type and Vmp^+^R) reacted to multiple proteins (representative samples for each group are shown in Fig. 4). In contrast, the serum samples from mice infected with Vmp^−^ spirochetes reacted to very few bands, and primarily to a single protein with a molecular mass of ∼20 kDa (Fig. 4), which was identified as Vtp in additional blots (data not shown). The serum from the Vmp^+^R-infected mouse #2 that had no detectable spirochetemia (Fig. 3) also had no serological response, confirming it was not infected (data not shown).

### Vmp^−^ mutant colonizes ticks like wild-type spirochetes

Ticks were infected with the wild-type, Vmp^−^, or Vmp^+^R strains to confirm the spirochetes’ ability to colonize the tick and move into the salivary glands. An RML mouse was first inoculated with 500 spirochetes of one of the strains and monitored by microscopy for peak spirochetemia. On day 4 the mice inoculated with wild-type and Vmp^+^R strains had high bacterial densities in the blood, at which time cohorts of *O. hermsi* ticks were fed on the mice and became infected. After 9 days spirochetes were still undetectable in the mouse inoculated with Vmp^−^, therefore another mouse was inoculated with 1.5×10^8^ spirochetes. The following day a high density of spirochetes in the blood was observed and a cohort of ticks was fed on the mouse. The quantity of bacteria in the blood was determined by QPCR with blood taken from the mice prior to tick feeding. The three mice used to infect the ticks with wild-type, Vmp^−^, and Vmp^+^R spirochetes had bacterial densities of 4.5×10^7^, 1.6×10^8^, and 2.1×10^7^cells/ml of blood, respectively.

Six ticks from each of the three groups were dissected 98–105 days after the ticks had fed and subsequently molted. Double-labeled IFA examinations demonstrated that all 18 ticks were infected with comparable numbers of spirochetes, which in the salivary glands produced Vtp (data not shown). Therefore, the inability of *B. hermsii* to make a Vmp did not prevent the spirochetes from colonizing the tick salivary glands.

### Vmp^−^ mutant does not cause a relapse in mice infected by tick transmission

**Figure 5:**
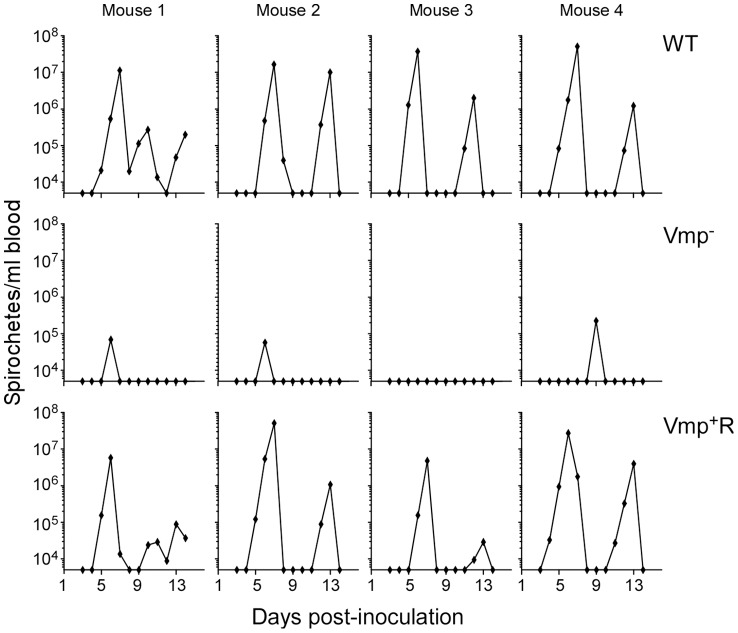
The *vmp* expression site is required for *B. hermsii* to relapse in mice infected by ticks. Ten ticks infected with either the wild-type (WT), Vmp^−^ or Vmp^+^R strains were placed on a mouse and allowed to feed. Mice were sampled on days 3–14 post infection and the numbers of spirochetes per ml of blood were determined by QPCR. Each plot represents the data from an individual mouse.

To test if the Vmp^−^ mutant can cause a relapse when transmitted by tick bite, four RML mice were each fed upon by 10 ticks infected with wild-type, Vmp^−^ or Vmp^+^R and monitored for infection. Spirochete concentrations were quantified in the blood by QPCR on days 3–14 post-feeding. All 4 mice fed upon by ticks infected with wild-type or Vmp^+^R developed an initial spirochetemia followed by a relapse (Fig. 5). In contrast, three of the four mice fed upon by ticks infected with Vmp^−^ showed an initial spirochetemia but no relapse was detected. Again, a strong serological response was detected in the mice infected with the wild-type or Vmp^+^R strains (data not shown), but the mice infected with the Vmp^−^ mutant showed a much reduced serological response, similar to what was observed with the needle-inoculated mice. These observations suggest that the Vmp^−^ mutant was likely cleared after the first spirochetemia.

### Spirochetemic levels of the Vmp^−^ mutant in mice are significantly reduced

**Figure 6:**
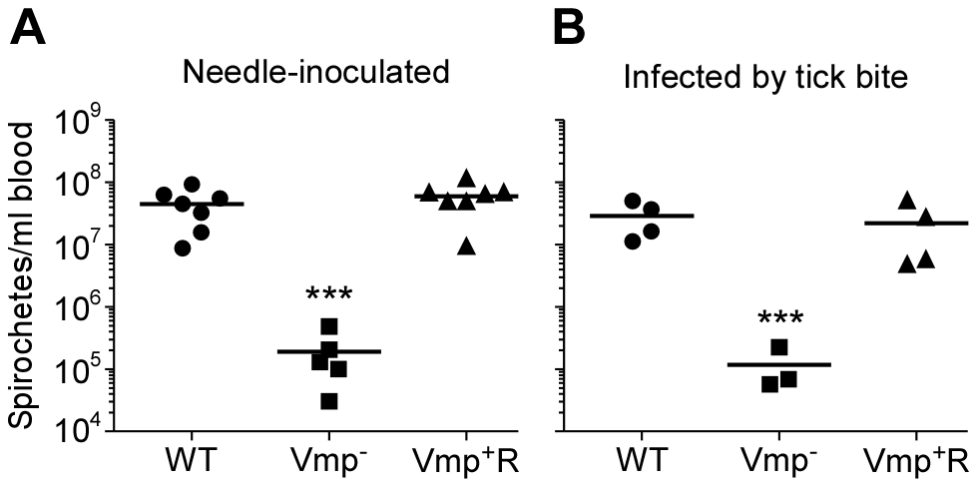
The spirochetemic levels of the Vmp^−^ mutant are significantly less than wild-type and Vmp^+^R strains. Each data point represents the number of spirochetes per ml of blood for an individual mouse during the first initial peak in needle-inoculated RML mice (n = 7 for Wild-type and Vmp^+^R, n = 5 for Vmp^−^) (**A**). First spirochetemic peak densities in RML mice infected by tick bite (10 ticks/mouse; n = 4 for Wild-type and Vmp^+^R, n = 3 for the Vmp^−^) (**B**). These data were taken from the first peaks shown in Fig. 3 & 5. The spirochetemic levels of Vmp^−^ mutant were significantly less than the levels of wild-type and Vmp^+^R strains (*** *P* < 0.0001). Statistical significance was determined by one-way ANOVA with the Tukey’s Multiple Comparison Test.

Not only was the Vmp^−^ mutant unable to cause a relapse in the mice, the levels of spirochetemia in the mice infected with Vmp^−^ were significantly reduced compared to the first spirochetemic peaks in the mice infected with wild-type and Vmp^+^R, regardless whether they were infected by needle-inoculation (Fig. 6A) or infected by tick bite (Fig. 6B). The Vmp^−^ spirochetes only reached a density as high as 10^4^–10^5^ cells/ml in the mouse blood, whereas the wild-type and Vmp^+^R strains reached 10^6^–10^8^ spirochetes/ml in the blood. This difference in the highest cell densities observed for the wild-type and Vmp^−^ spirochetes was not explained by monitoring their in vitro growth. These two strains had identical growth curves in culture counted for five consecutive days, with the wild-type and Vmp^−^ mutant spirochetes achieving cell densities of 1.66×10^8^ and 2.88×10^8^ per ml, respectively.

### Vmp^−^ mutant produces Vtp during its spirochetemic peak

**Figure 7:**
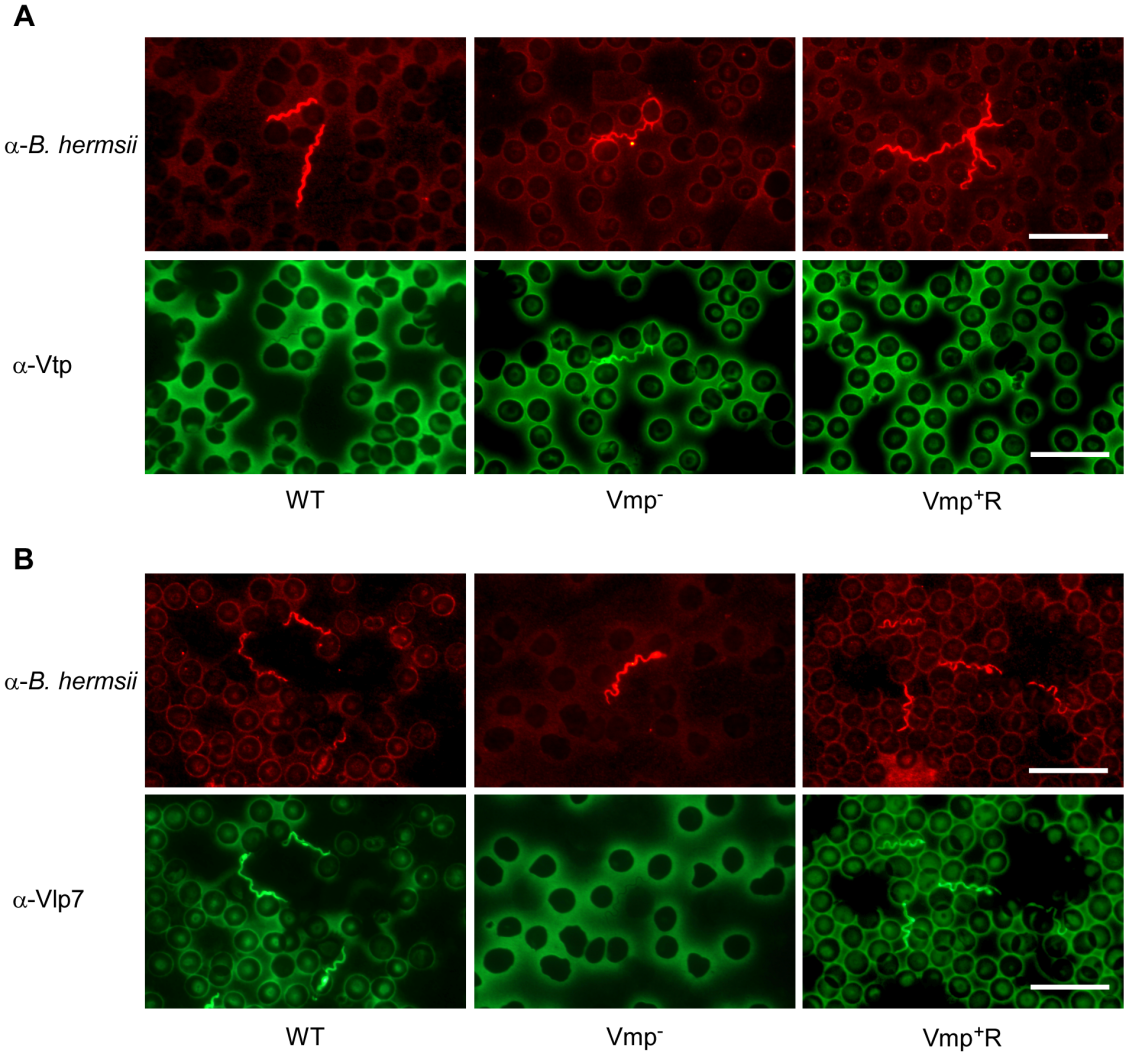
The Vmp^−^ mutant *B. hermsii* produces Vtp during its spirochetemia. Spirochetes in blood smears collected from mice infected with the wild-type (WT), Vmp^−^ mutant or reconstituted (Vmp^+^R) strains were double-labeled with a rabbit anti-*B. hermsii* hyper-immune serum and a mouse anti-Vtp (**A**) or anti-Vlp7 (**B**) monoclonal antibody. The wild-type and Vmp^+^R spirochetes produced Vlp7 during the first spirochetemic peak in mice while the Vmp^−^ mutant produced Vtp.

Since the Vmp^−^ mutant produced Vtp in culture, we asked whether this protein was produced in the blood during the spirochetemic peak. IFAs performed with monoclonal antibodies to Vtp and Vlp7 showed that the mutant produced Vtp but not Vlp7 in mice infected by needle inoculation (Fig. 7) or infected by tick bite (data not shown). In all mice infected with the wild-type or reconstituted strains, the spirochetes in the first spirochetemic peak produced Vlp7 (Fig. 7B) but not Vtp (Fig. 7A).

### Vmp^−^ mutant persists in SCID mice but at lower cell densities than wild-type spirochetes

**Figure 8:**
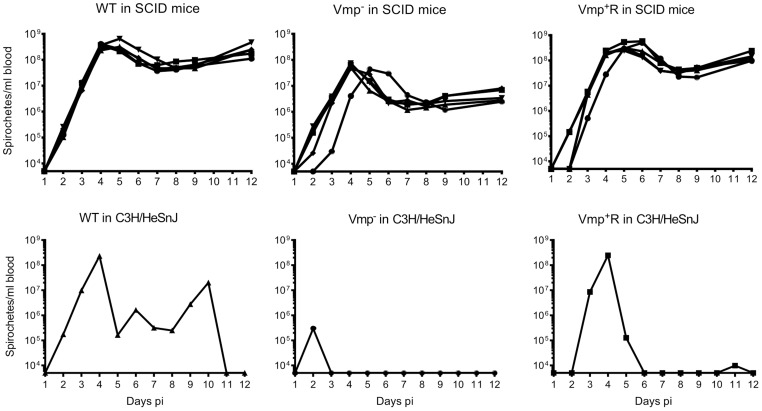
The Vmp^−^ mutant *B. hermsii* persists in the blood of SCID mice. Five SCID mice and 1 wild-type mouse with the same genetic background (C3H/HeSnJ) were injected ip with 500 spirochetes of wild-type (WT), Vmp^−^, or reconstituted (Vmp^+^R) strains. Mice were sampled on days 1–12 post-infection and the numbers of spirochetes/ ml of blood were determined by QPCR. Growth rates of the three strains in SCID mice during three consecutive days of exponential growth were not significantly different based on linear regression analysis of the slopes (*P* = 0.1129).

**Figure 9:**
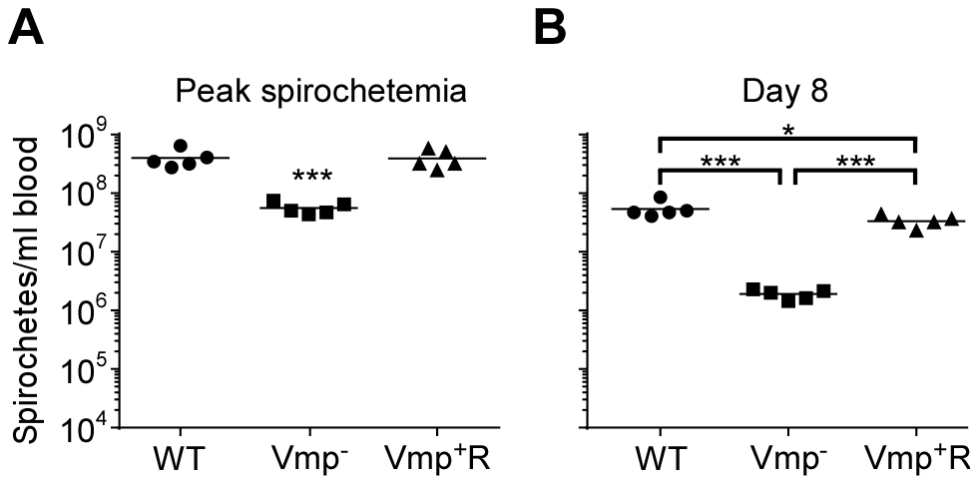
The Vmp^−^ mutant *B. hermsii* has reduced spirochetemic levels in the blood of SCID mice. Each data point represents the number of spirochetes per ml of blood for an individual mouse during the first spirochetemic peak (**A**) and later during a persistent infection (day 8) in SCID mice (**B**) (n = 5 mice for each strain). These data were taken from two time points presented in Fig. 8. The spirochetemic levels of Vmp^−^ mutant were significantly less than the wild-type (WT) and reconstituted (Vmp^+^R) strains (*** *P* < 0.0001, *P < 0.05). Statistical significance was determined by one-way ANOVA with the Tukey’s Multiple Comparison Test.

To address whether the lower cell densities and quick disappearance of the Vmp^−^ mutant was due more to the innate immune response than to the adaptive immune response, we infected SCID mice with the 3 strains. Five SCID mice and 1 immunocompetent C3H/HeSnJ mouse were inoculated i.p. with 500 wild-type, Vmp^−^, or Vmp^+^R spirochetes and the bacterial densities in the blood were determined by QPCR on days 1–12 post-inoculation. In the wild-type C3H/HeSnJ mice, the spirochetes had similar spirochetemia patterns to those in RML mice. The wild-type and Vmp^+^R strains produced an initial spirochetemia and a relapse, whereas the Vmp^−^ spirochetes were unable to relapse (Fig. 8). Also, the mutant did not achieve as high cell densities as the wild-type and reconstituted strains (10^5^ versus 10^8^, respectively). Even though the level of wild-type spirochetes in the C3H/HeSnJ mouse never went below detection, IFAs performed on blood smears collected on days 4 and 10 determined that 100% of the spirochetes produced Vlp7 on day 4 in contrast to 0% on day 10, indicating a switch had occurred in this mouse and a clearance was missed in the sampling times. All 15 SCID mice remained persistently infected with the 3 strains (Fig. 8), although the wild-type and Vmp^+^R spirochetes achieved cell densities 7 times greater than the Vmp^−^ spirochetes at the peak spirochetemia (Fig. 9A). The mutant reached densities of 10^7^ cells/ml whereas wild-type and reconstituted strains reached densities of 10^8^ cells/ml. The level of spirochetemia of the Vmp^−^ mutant was considerably higher in SCID mice versus wild-type mice. However, throughout the infection, the mutant spirochete concentrations were consistently lower than the wild-type or Vmp^+^R spirochetes. For example, on day 8 (Fig. 9B), when the concentration of spirochetes in the blood of SCID mice appeared to have leveled, the wild-type densities were 28 times greater than the Vmp^−^ mutant with an average of 5.4×10^7^ versus 1.9×10^6^ cells/ml, respectively; Vmp^+^R was 17 times more abundant than Vmp^−^ with an average of 3.3×10^7^ cells/ml. IFAs performed on blood smears collected on day 12 from SCID mice showed that 100% of Vmp^−^ spirochetes were producing Vtp. In wild-type and Vmp^+^R spirochetes, 34–54% and 12–87%, respectively, were still producing Vlp7 on day 12 compared to day 3 when 90–100% were producing Vlp7. Also, on day 12 the SCID mice began showing signs of illness and the experiments were terminated. These observations show that the wild-type and reconstituted strains switched the *vmp* in the expression site without an acquired immune response in the host. In contrast, the Vmp^−^ mutant that was unable to produce a Vmp continued to produce Vtp.

### Vtp-minus mutant colonizes ticks but is not infectious by tick bite

**Figure 10:**
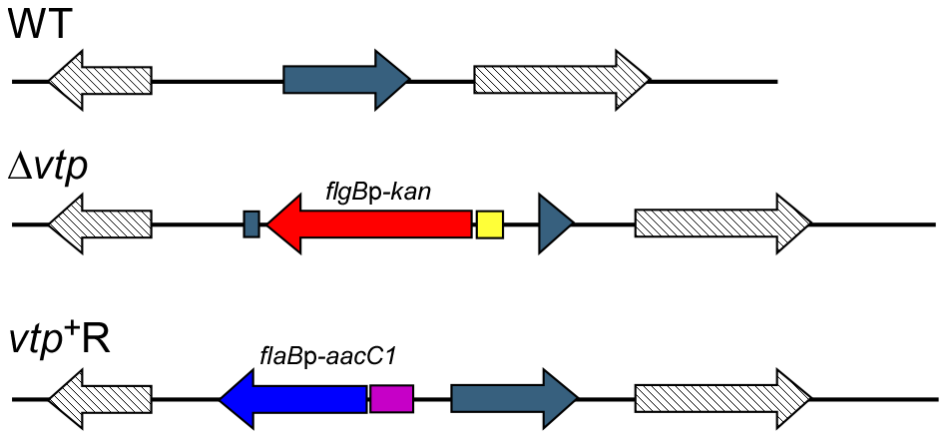
Diagram of the *vtp* locus in the wild-type, Δ*vtp* mutant and reconstituted strain *vtp* ^+^R. The *vtp* gene in the wild-type (WT) *B. hermsii* DAH was inactivated by deleting most of the gene and replacing it with the *flgB*p-*kan* cassette by homologous recombination, creating the Δ*vtp* mutant. The reconstituted strain (*vtp* ^+^R) was constructed by replacing the Δ*vtp* mutation with the *flaB*p-*aacC1* cassette next to an intact *vtp* gene, also by homologous recombination. Details were described previously [28].

**Figure 11:**
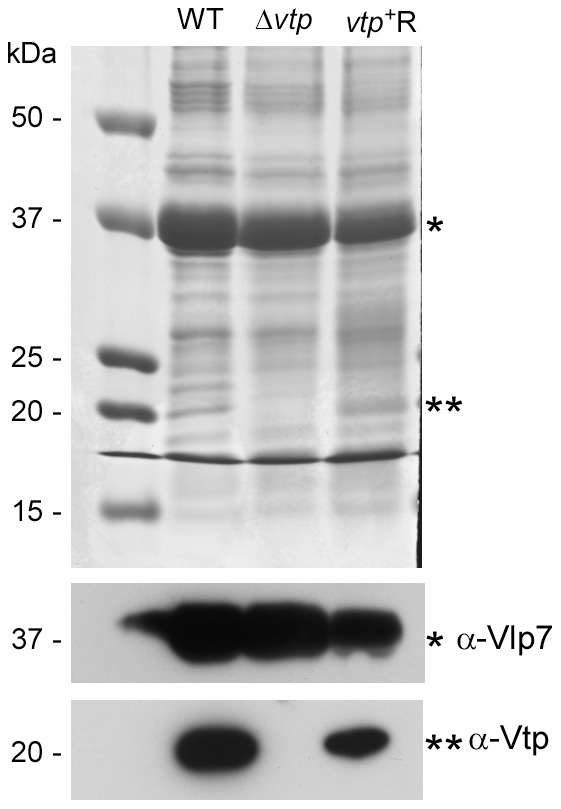
The Δ*vtp* mutant *B. hermsii* does not produce the variable tick protein. Coomassie-stained PAGE gel of whole cell lysates (top panel) of the wild-type (WT), Δ*vtp* mutant and reconstituted (*vtp* ^+^R) strains and immunoblots probed with anti-Vlp7 (middle panel) and anti-Vtp (bottom panel) monoclonal antibodies. All 3 strains produced Vlp7 while the mutant did not produce the variable tick protein (Vtp). Molecular mass standards are shown at left in kDa. * indicates Vlp7 protein, ** indicates Vtp protein.

When wild-type spirochetes are acquired by a feeding tick, the bacteria gradually down-regulate bloodstream Vmps and up-regulate Vtp. During persistent infection in the tick salivary glands, the spirochetes exclusively produce Vtp but quickly switch back to the Vmp phenotype when reintroduced to mammalian blood. Given the rapid temporal switch of *B. hermsii* from Vtp to Vlp7 during mammalian infection by tick-bite [23], and the ongoing production of Vtp by the Vmp^−^ mutant shown above, we tested an isogenic strain of the spirochete in which *vtp* was deleted (Δ*vtp*)(Fig. 10, modified from Battisti et al.) [28], which rendered the spirochete unable to produce Vtp (Fig. 11). Synthesis of Vtp during persistent infection of the tick salivary glands suggested that this protein might be required by *B. hermsii* for migration to the salivary glands, stable tick infection, or initial colonization in mammals. To test these hypotheses, we used genetically transformed spirochetes described previously [28], in which the *vtp* gene was inactivated (Δ*vtp*) in *B. hermsii* and the mutant reconstituted with the wild-type *vtp* gene (*vtp* ^+^R). Battisti and colleagues previously showed that the Δ*vtp* mutant remained infectious in mice by needle inoculation [28], as these spirochetes continued to produce bloodstream Vmps in vitro. In this study we compared these isogenic spirochetes with wild-type *B. hermsii* in experimental infections with *O. hermsi* ticks and mice.

Ticks were infected with wild-type, Δ*vtp*, or *vtp* ^+^R *B. hermsii* by feeding them on bacteremic mice and were examined by microscopy for spirochetes producing Vtp in the midgut and salivary glands at various times after their acquisition (Table 1). No wild-type *B. hermsii* were detected in the salivary glands on day 7, and only 1 of 5 ticks had a low level infection in the glands on day 14. By day 21, all ticks had salivary gland infections but only 6.8% of the wild-type spirochetes in these tissues were producing Vtp. During the ensuing months, however, over 90% of the spirochetes became Vtp+, decreasing to 76% by day 448.

**Table 1:** Percent *Borrelia hermsii* Vtp+ in tick midgut and salivary glands.

	Midgut				Salivary Glands			
Days PI	No. Positive MG^a^/5	No. Spirochetes examined	No. Spirochetes Vtp+	% Spirochetes Vtp+	No. Positive SG^b^/5	No. Spirochetes examined	No. Spirochetes Vtp+	% Spirochetes Vtp+
Wild-type								
7	5	500	0	0	0	NA	NA	NA
14	5	262	0	0	1	5	0	0
21	5	295	20	6.8	5	103	7	6.8
35	5	183	29	21	5	379	220	58
49	5	331	5	1.5	5	153	85	55.6
116	5	52	9	17.3	5	232	214	92.2
221	0	NA	NA	NA	4	124	120	96.8
448	1	41	1	2.4	4	237	180	75.9
Totals	31/40	1664	64	3.8	29/40	1233	826	67
Δ*vtp*								
7	5	500	0	0	0	NA	NA	NA
14	5	500	0	0	1	57	0	0
21	5	279	0	0	5	56	0	0
35	5	245	0	0	5	608	0	0
47	5	256	0	0	5	361	0	0
111	1	4	0	0	5	64	0	0
217	0	NA	NA	NA	4	42	0	0
443	2	88	0	0	4	278	0	0
Totals	28/40	1872	0	0	29/40	1466	0	0
*vtp* ^+^R								
49	5	391	10	2.6	5	231	212	91.8

a MG denotes midgut.

b SG denotes salivary glands.

Ticks infected with Δ*vtp B. hermsii* were examined 7 to 443 days after feeding (Table 1). These spirochetes also disseminated from the midgut to the salivary glands and persisted in these tissues out to 443 days after tick feeding. However, none of 1,466 and 1,872 mutant spirochetes observed in the salivary glands and midgut, respectively, was Vtp+. These results demonstrate that the synthesis of Vtp was not required for *B. hermsii* to escape the tick’s midgut or to colonize and persist in the salivary glands.

Additional ticks were infected with the reconstituted *vtp* ^+^R spirochetes. These ticks were dissected 49 days after feeding and spirochetes in the midgut and salivary glands were examined for Vtp (Table 1). Like the wild-type spirochetes, the *vtp* ^+^R *B. hermsii* also disseminated from the midgut to the salivary glands and up-regulated the synthesis of Vtp during tick infection.

Spirochetes were also examined in a few ticks from day 7 to 116 days after infection for the presence of Vlp7, which was the Vmp produced when acquired by the ticks (5 ticks infected with wild-type and 6 ticks infected with Δ*vtp* mutant spirochetes) (Table 2). No spirochetes were Vlp7+ in the salivary glands (0/254) while 63% (284/452) of spirochetes were Vlp7+ in the midgut. For both *B. hermsii* strains these results confirmed our earlier observations regarding the partitioning of phenotypes, with no spirochetes producing bloodstream Vmps in the tick salivary glands [23].

**Table 2:** Percent *Borrelia hermsii* Vlp7+ in tick midgut and salivary glands.

		Midgut			Salivary Glands		
Strain	No. ticks	No. Spirochetes examined^a^	No. Spirochetes Vlp7+ b ^,^ c	% Spirochetes Vlp7+	No. Spirochetes examined	No. Spirochetes Vlp7+	% Spirochetes Vlp7+
Wild-type	5	147	65	44	170	0	0
Δ*vtp*	6	305	219	72	84	0	0

a Ticks were infected by feeding on spirochetemic mice and examined 7–116 days after infection.

b Number of spirochetes producing Vlp7 detected by double-labeled IFA.

c The number of spirochetes producing Vlp7 in the midgut versus the salivary glands is significantly different (Fisher’s Exact Test, *P*<0.0001).

Transmission experiments were performed with ticks infected with wild-type, Δ*vtp* mutant or *vtp* ^+^R spirochetes on days ranging from 66 to 386 after their previous infectious blood meal. All mice (100%) fed upon by ticks infected with wild-type or *vtp* ^+^R *B. hermsii* developed spirochetemias detectable by microscopy and were later seropositive (Table 3). In contrast, none of the mice fed upon by the Δ*vtp B. hermsii*-infected ticks became spirochetemic or seroconverted. Immunological staining of the spirochetes in the blood smears from the 10 mice that developed detectable spirochetemias, identified the bacteria as serotype 7, which again was the same serotype that the ticks were infected with previously. These results demonstrate that Vtp is required for mammalian infectivity of *B. hermsii* by tick bite.

**Table 3:** Vtp mutant is non-infectious to mice by tick bite.

Strain	No. Mice	No. Spirochetemic^a^	No. Seropositive^b^	% Infected	1^st^ Serotype
Wild-type	6	6	6	100	7
Δ*vtp* c	6	0	0	0	NA
*vtp* ^+^R	4	4	4	100	7

a No. of mice spirochetemic within 10 days post-infection.

b No. of mice seropositive examined 3–4 months post-infection.

c The lack of infection by the mutant compared to the wild-type and reconstituted strains is significantly different (Fisher’s Exact Test, *P* = 0.0001).

## Discussion

In this study, we examined the necessity of switching between Vmps or Vtp during the life cycle of *B. hermsii.* Evading the host’s humoral immune response would allow repeated high levels of bacteremia, which clearly increases the potential for these spirochetes to be acquired by their fast-feeding tick vector that ingests very small volumes of blood [15], [35]. However, the adaptive significance of the spirochete’s switch from a bloodstream Vmp to Vtp while in the tick for transmission back to a mammal is far less intuitive. The switch is even more perplexing, given that the spirochetes retain the same Vmp gene in the expression site while infecting ticks, and that spirochetes producing Vmps and not Vtp are infectious when passaged from one mouse to another by needle inoculation of infected blood [4]. Phenotypes of the *vmp* and *vtp* deletion mutants of *B. hermsii* shed light on the biological significance of replacing a bloodstream Vmp with Vtp when *B. hermsii* resides in ticks.

### Infection in immunocompetent mice

The *B. hermsii* Vmp^−^ mutant was attenuated in immunocompetent mice when inoculated by needle or delivered naturally by tick-bite. The attenuation was characterized by 1) fewer mice with a detectable spirochetemia, 2) reduced cell density during the initial spirochetemia, and 3) no mice with a detectable relapse. The much reduced antibody response observed after infection with the Vmp^−^ mutant was consistent with the transient, low-level spirochetemia.

The inability of the *B. hermsii* Vmp^−^ mutant to relapse in immunocompetent mice was our predicted outcome for these infection experiments. Our study is the first to identify host-associated phenotypes in the life cycle of a relapsing fever spirochete by genetically inactivating specific gene targets. Lyme disease spirochetes *Borrelia burgdorferi* also contain an elaborate mechanism for antigenic variation first described by Zhang and colleagues [36]. The expression site is telomeric on linear plasmid lp28-1 and contains a *vmp*-like sequence, designated *vlsE,* which has highest sequence similarity to *vlp17* of *B. hermsii*. Upstream of the *vlsE* expression site are 15 silent cassettes that contain internal variable sequences, portions of which recombine into *vlsE* with the potential to create millions of unique *vlsE* alleles [36]. One striking difference between these two species of spirochetes is that the *vls*E locus in *B. burgdorferi* produces an extremely high number of antigenic variants in a single host at one time, such that the spirochetes have the ability to continually evade the host’s humoral immune response and persist throughout the life of the infected animal [37]. Experimental infections with *B. burgdorferi* show that laboratory mice remained infected for a year [38], [39] to 16 months after inoculation until the animals were euthanized [40]. Infected white-footed mice (*Peromyscus leucopus*), a primary reservoir for *B. burgdorferi* in eastern North America [41], [42], continually infected new cohorts of ticks in the laboratory for many months [43], and are likely capable of doing so for their entire life once infected.

Evidence for the possible role of *vlsE* for persistent infection of *B. burgdorferi* in mammals was demonstrated with clones lacking lp28-1, the plasmid encoding this locus, which had a reduced (“intermediate”) infectivity in mice [30], [36], [44]. Subsequently, Bankhead and Chaconas [45] demonstrated that clones of *B. burgdorferi* lacking the *vlsE* locus via targeted genetic inactivation were present in blood of mice on days 4 and 7 postinoculation but were no longer isolatable from any mouse tissues by 3 weeks. That study demonstrated the essential role of the *vlsE* locus for *B. burgdorferi* to persistently infect mammals. Thus, both *B. hermsii* and *B. burgdorferi* have linear plasmid-encoded expression loci that are essential for each spirochete’s production of antigenic variants to persist for either shorter (weeks to a few months) or longer (life of the host) periods of time, respectively.

Another objective of our study was to elucidate additional functions that the Vmps might provide the spirochete other than to evade the host’s humoral immune response. During the first detectable peak in spirochetemia, the Vmp^−^ mutant achieved a significantly lower mean cell density in the blood at approximately 10^5^ spirochetes per ml whereas wild-type and reconstituted strains were more abundant at 10^7^ to 10^8^ spirochetes per ml (Fig. 6). Because peaks in spirochetemia of the mutant coincided with the same days for the peaks in spirochetemia for mice infected with the wild-type and reconstituted spirochetes (Fig. 3 & 5), we believe that the differences we observed in spirochete densities were not due to different growth rates but rather a reduced fitness of the Vmp^−^ mutant. This hypothesis was supported by nearly identical growth curves of the wild-type and Vmp^−^ mutant spirochetes when grown in culture and SCID mice.

In the normal transmission cycle, *B. hermsii* down-regulates the bloodstream Vmp following its acquisition by ticks, and up-regulates Vtp while persistently infecting the tick’s salivary glands (this report and [23]). During the tick’s subsequent feeding, Vtp-positive spirochetes are transmitted to the mammalian host, but by 3 to 5 days post infection (when the spirochetes are abundant enough to be visualized by microscopy), the spirochetes no longer make Vtp and have switched back to the same bloodstream Vmp that the spirochetes were making when previously acquired from the infected host [23]. In our infection experiments with immunocompetent mice, the phenotypes of wild-type and Vmp^+^R reconstituted spirochetes were as expected; borreliae in the first spirochetemia produced Vlp7 but not Vtp (Fig. 7). However, the Vmp^−^ mutant remained Vtp-positive during its initial and only detectable peak of infection in immunocompetent mice. Might the prolonged synthesis of Vtp, and the spirochetes’ inability to switch back to Vlp7 confer a disadvantage during growth in vivo, separate from evading an antibody response directed to Vtp by the infected host?

The presence of Vtp-positive cells in the Vmp^−^ mutant population during early infection in immunocompetent mice was not totally unexpected. Barbour et al. [26] demonstrated that the synthesis of Vtp is controlled at the level of transcription, and when a *vmp* gene is transcribed in vitro, *vtp* is not. The reverse is also true such that *B. hermsii* cells express a *vmp* or *vtp*, but not both genes simultaneously [26]. Therefore, we hypothesized that our *B. hermsii* Vmp^−^ mutant would constitutively produce Vtp, which is what we observed.

The spirochetemia plots displayed by our Vmp^−^ mutant that produced Vtp showed an initial reduced cell density and no relapse (Fig. 3 & 5). This pattern of infection is strikingly similar to the infection profiles reported by Alugupalli et al. [9], who used the same strain of *B. hermsii* that we used (DAH) but after the culture was passaged 19 times in vitro prior to inoculation in mice. *B. hermsii* does not typically lose plasmids and infectivity during in vitro cultivation [46], [47] as does *B. burgdorferi* [44], [48], [49], however, the continuous growth in vitro appears to attenuate *B. hermsii* similar to what we observed with the low passaged Vmp^−^ mutant. Although Alugupalli et al. [9] did not identify the serotype of *B. hermsii* after 19 passages, we assume that the majority, if not all, of the spirochetes had switched to Vtp (previously named “culture type” and Serotype C [4], [50] and pIc, VmpC, and Vsp33 [25], [51]). Barbour et al. [26] also observed that two culture-derived serotype C isolates of *B. hermsii* HS1 were less virulent in irradiated mice compared to lower passaged cultures comprised of other serotypes. One possible explanation for wild-type *B. hermsii* becoming attenuated during continuous growth in culture might be the loss of efficient regulation for switching the expression of *vtp* to a *vmp* when spirochetes are inoculated into a mammal. Our attenuated Vmp^−^/Vtp+ mutant stimulated an antibody response in the immunocompetent mice that was restricted almost entirely to Vtp (Fig. 4). Additionally, we have produced antibodies to purified Vtp in rabbits that are borreliacidal to *B. hermsii* cells producing Vtp (Schwan, unpublished observations). Therefore, we believe that the Vmp^−^ mutant stimulated the production of neutralizing antibodies to Vtp that quickly cleared the infection in mice.

### Infection in SCID mice

The antigenically distinct serotypes of *B. hermsii* and other relapsing fever spirochetes stimulate a specific IgM antibody response produced by the host’s B1b lymphocytes, which results in the clearance of the majority of the spirochetes from the blood [9], [10], [12], [52]. Rare spontaneous antigenic variants that are unique from the infecting serotype escape the antibody-mediated killing and are progenitors for new serotypes to repopulate the blood [4]. In the absence of a host’s antibody response, our predictions were that the infecting serotype of *B. hermsii* would not be cleared and instead maintain a persistently high cell density in the blood. Also, with no antibody-based immune pressure to select a new serotype, the wild-type spirochetes might not switch Vmps. Regarding our first prediction, we observed what other investigators have demonstrated previously; *B. hermsii* achieves persistently high levels of bacteremia in irradiated, SCID or chemically-treated mice deficient in their production of antibodies [4], [9], [53], [54]. All three isogenic strains of *B. hermsii* (wild-type, Vmp^−^ mutant, Vmp^+^R) produced high and persistent spirochetemias in SCID mice for 12 days after needle inoculation (Fig. 8). However, immunofluorescent stains of the wild-type and reconstituted spirochetes demonstrated that, while early in their infections (Day 3) nearly 100% of the spirochetes were Vlp7-positive (the infecting serotype), by Day 12 the percentage of spirochetes that were Vlp7-positive had dropped significantly to as low as only 12% in one of the mice. Therefore, just as Stoenner et al. [4] observed the spontaneous conversion to variant *B. hermsii* in cyclophosphamide-treated mice and in the fortified Kelly’s medium [4], our serotype 7 spirochetes also switched to other unidentified serotypes during infection in SCID mice.

The Vmp^−^ mutant showed significantly lower levels of spirochetemia in SCID mice compared to the wild-type and reconstituted spirochetes (Fig. 8 & 9), just as we observed with the infections in immunocompetent mice (Fig. 3 & 5). The Vmp^−^ mutant remained Vtp-positive during the 12 days of infection, unlike the wild-type and reconstituted spirochetes that switched spontaneously from Vlp7 to other Vmps. Although the Vmp^−^ mutant/Vtp-positive spirochetes persisted in the absence of antibody, these bacteria were moderately attenuated in the SCID mice, in that they were unable to grow to the same cell densities. Thus the Vmp^−^ mutant *B. hermsii* was less fit than wild-type and reconstituted spirochetes even in immunodeficient mice. How might the spirochete’s inability to produce a Vmp create a disadvantage for their growth in vivo? Alugupalli et al. [9] observed a reduction in the density of *B. hermsii* cells during persistent infection in SCID mice as did we, and these investigators suggested that some arm of the host’s innate immunity might elicit some control of the spirochetemia. If such an impact of innate immunity is involved, then possibly the Vmps of *B. hermsii* offer slightly greater protection to the cells than does Vtp.

The reduced cell density of the Vmp^−^ mutant in the blood also suggests a possible growth defect in nutrient acquisition. *B. hermsii* and other species of relapsing fever spirochetes have pathways for the uptake and utilization of glycerol and purines that are absent in Lyme disease spirochetes [55]–[57]. We have speculated previously that these additional metabolic pathways in the relapsing fever spirochetes might help these bacteria to achieve much higher cell densities in the blood than do the Lyme disease spirochetes [56], [57]. Possibly the presence of a Vmp rather than Vtp on the spirochete’s outer surface allows for the greater uptake of glycerol, the salvage of purines, or the uptake of other nutrients in the blood (while also offering some protection from the host’s innate immunity). Another scenario might be a difference in rate of bacterial clearance in the spleen and liver due to spirochetes with different abundant outer surface proteins having different affinities for binding to the cellular components of the blood. *B. hermsii* cells bind to platelets and erythrocytes [53], [58], [59] and the presence of Vtp rather than the bloodstream Vmps might make the spirochetes more adherent to cells and more susceptible to their removal in the spleen and liver [58]. While the mechanism(s) are not known, Vmp-positive *B. hermsii* had an advantage over the Vmp^−^ mutant/Vtp-positive cells in mice, which suggests that the variable major proteins confer some advantage to the spirochetes separate from contributing to a mechanism of immune evasion.

### Vmp and VlsE production in ticks

*B. hermsii* and *B. burgdorferi* differ in their population of variants that are present in a single host [37]. A multitude of *B. burgdorferi* VlsE variants are present simultaneously in the mouse while the vast majority of *B. hermsii* cells present at one time are comprised of one serotype that produce the same Vmp [60]. Given the importance of variation in the Vmp and VlsE antigens to prolong mammalian infection via immune evasion, the lack of change in these surface proteins in ticks might be expected and is what we observed. In all acquisition and transmission events that we monitored with wild-type and reconstituted spirochetes, *O. hermsi* ticks transmitted spirochetes of the same serotype (i.e., no change in Vmp) that they acquired during their previous blood meal. Therefore, no recombination events occurred in the *vmp* expression site during infection in ticks. Additionally, many wild-type *B. hermsii* no longer produced Vlp7 when in the tick midgut and no Vlp7-positive spirochetes were found in the tick salivary glands. When *B. burgdorferi* infects its tick vector *Ixodes scapularis*, genetic variation in the VlsE locus also ceases and spirochetes quickly shut down the production of the protein [61]–[64]. Cumulatively, these observations demonstrate that neither wild-type *B. hermsii* nor *B. burgdorferi* change Vmp or VlsE antigens while infecting their respective tick vectors. Our infections with Vmp^−^ mutant *B. hermsii* in *O. hermsi* also demonstrated conclusively that this spirochete’s mechanism to vary antigenically in mammals was not required to colonize and persist in ticks. Additionally, the Vmp^−^ mutant still produced Vtp in the tick salivary glands, and was infectious, although attenuated, in mice when subsequently transmitted by tick bite. We are not aware of a comparable study in which a specific *vlsE* ^−^ mutant *B. burgdorferi* has been tested for infectivity when transmitted by its hard tick vector.

### Vtp required for infectivity by tick bite

The *B. hermsii Δvtp* was the second deletion mutant we examined in the infectious cycle with ticks and mice. Previous work in our laboratory [23] and the new time-course data presented here (Table 1) demonstrate that wild-type *B. hermsii* up-regulated Vtp synthesis in ticks and that the switch was gradual over many weeks, as spirochetes disseminated from the midgut to salivary glands. Over 90% of the spirochetes in the salivary glands became Vtp-positive while relatively few bacteria that remained in the midgut made the switch. The presence of wild-type *B. hermsii* in the tick salivary glands that had not yet begun to produce Vtp suggested this protein was not required for the spirochetes to either escape from the midgut or colonize the salivary glands. This hypothesis was confirmed with the *Δvtp* mutant, which was able to colonize and persist in the tick salivary glands for well over a year (63 weeks) (Table 1).

The striking phenotype of the *Δvtp* mutant was its loss of infectivity when ticks infected with this spirochete fed on mice. None of the mice exposed cumulatively to 60 ticks infected with this mutant became spirochetemic or seroconverted, while all mice fed upon by ticks with the wild-type (60 ticks) and reconstituted spirochetes (34 ticks) became infected. Therefore, synthesis of Vtp by *B. hermsii* while infecting the salivary glands is essential for spirochete infectivity in mice when delivered by tick bite. Our results are strikingly similar to studies that have examined the role of the Vtp orthologous protein, OspC, produced by *B. burgdorferi* during its infectious cycle in ticks and mammals. This major outer surface protein is not produced by spirochetes in the midgut of unfed *Ixodes scapularis* ticks. But by 48 hr after ticks have attached to a warm-blooded mammal and begun to feed, spirochetes turn on the synthesis of OspC [65]–[68]. Production of OspC is tightly coordinated with the transmission of *B. burgdorferi* while ticks feed, however, this protein is not required for spirochetes to escape the midgut and penetrate the salivary glands [69]. However, OspC is essential for *B. burgdorferi* to be infectious in mammals via tick bite [69], just as we have observed with our *Δvtp* mutant. Thus, both *B. hermsii* and *B. burgdorferi* require the synthesis of the orthologous proteins Vtp and OspC, respectively, to infect mammals by tick bite, yet the essential roles provided by these proteins are unknown.

The mechanism underlying the regulatory control of OspC synthesis by *B. burgdorferi* during tick feeding has received much attention and was nicely reviewed by Samuels [70]. From the initial observations that an increase in temperature and the tick’s blood meal stimulate the upregulation of OspC [65], [66], [71], the production of this protein and many others requires the Rrp2-RpoN-RpoS cascade. Little is yet known about the regulatory control of Vtp by *B. hermsii* but two interesting differences are apparent. First, while an increase in temperature during in vitro growth stimulates *B. burgdorferi* to make OspC, the opposite is true for *B. hermsii*, which turns on Vtp when grown in culture at lower temperatures and following acquisition from a warm-blooded mammal to the cold-blooded tick [23]. These opposite affects of temperature may relate to bacterial growth rate as was recently proposed for *B. burgdorferi* [72]. We have recently released the genomes of four isolates of *B. hermsii*, which are available in GenBank of the National Center for Biotechnology Information. These data include the chromosomal sequence of the isolate *B. hermsii* DAH (CP000048) we used in our present study, as well as *B. hermsii* YOR (CP004146), *B. hermsii* MTW (CP005680) and *B. hermsii* YTB (CP005706). While these spirochetes contain most genes of the two component regulatory response-RpoS cascade, none of them contains an intact RpoN gene. Therefore, comparative differences in how *B. burgdorferi* and *B. hermsii* control the synthesis of OspC and Vtp, respectively, await further investigation.

Interestingly, the reciprocal expression of a *vmp* and *vtp* by *B. hermsii* while grown in vitro, in which single cells produce either a Vmp or Vtp, but not both [26] was not what we observed in the salivary glands of infected ticks. Our double-label IFA stains demonstrated that spirochetes never produced Vlp7 in tick salivary glands regardless of the strain examined. The analysis of the Δ*vtp* mutant showed that these *B. hermsii* cells lacked both Vtp and a Vmp in the salivary glands and were not infectious by tick bite. Recently, Marcisisin and colleagues described such a *B. hermsii* variant that arose spontaneously in the type strain HS1, which after years of continuous in vitro cultivation no longer produced a Vmp or Vtp [73]. This spirochete was no longer infectious in mice by needle inoculation but was used to hyperimmunize animals to generate antibodies to other surface proteins. Our *Δvtp* mutant remained infectious by needle inoculation, which we assume was due to the production of Vlp7 during growth in vitro.

Our *B. hermsii* Vmp^−^ mutant constitutively produced Vtp while infecting mice, but the strain was attenuated and cleared by mice capable of producing an antibody response to infection. Additionally, Vtp was required for spirochetes to be infectious by tick bite but the constitutive presence of this protein made the spirochetes less fit. Among the approximately 75 isolates of *B. hermsii* that we have established during the last 25 years from human patients, wild mammals and ticks, we have identified 9 antigenically distinct groups of Vtp proteins, which between the groups share only 60 to 75% amino acid identity, yet within each group the sequences are identical or nearly so [27](unpublished data). This polymorphism in Vtp suggests there is a selective pressure driven by the natural hosts’ immune response, which has been suggested to be the driving force for polymorphism of OspC types found among isolates of *B. burgdorferi* [74], [75].

*B. hermsii* spirochetes quickly switch from Vtp to a Vmp after transmission by tick bite. While we do not know the function that makes Vtp essential for vector-borne infection, we suggest the following speculative model for the adaptive value for this spirochete to enter a host body with one antigenic type (Vtp) and then quickly switch to another (Vmp). If spirochetes first colonize the blood stream of the host while producing Vtp, which starts to stimulate an IgM antibody response to this surface protein, but then quickly change to Vmp-producing cells via the switch between two promoters [26], the infecting population may have a growth advantage that allows the cells to achieve a higher cell density. *B. hermsii* cells producing Vtp upon entry into the host might temporarily divert the acquired immune response to a surface protein that quickly disappears. This could allow spirochetes to establish the infection, replicate, switch and possibly achieve a higher cell density in the blood before being cleared. An adaptation that results in a delay in the host’s immune response to clear the infecting serotype would confer an advantage for their acquisition by tick bite by allowing more spirochetes to circulate in the blood at a higher cell density. We hope to test this hypothesis in the future with *B. hermsii* that produce a bloodstream Vmp in place of Vtp when transmitted by ticks to mammals.

## Materials and Methods

### Ethics statement

The Rocky Mountain Laboratories, NIAID, NIH, Animal Care and Use Committee approved study protocols #2009-32, #2009-87, #2012-29 and #2012-70 for the feeding of ticks on mice, infecting mice with spirochetes, and the isolation of spirochetes from mouse blood samples. All work in our study was conducted adhering to the institution’s guidelines for animal husbandry, and followed the guidelines and basic principals in the Public Health Service Policy on Humane Care and Use of Laboratory Animals, and the Guide for the Care and Use of Laboratory Animals, United States Institute of Laboratory Animal Resources, National Research Council.

### Bacterial strains and growth conditions

*B hermsii* DAH 2E7 was cloned by limiting dilution in liquid mBSK-c medium from the low-passaged non-clonal isolate DAH [18], [27], [28], [76]. Although much of the studies regarding antigenic variation in *B. hermsii* have been done with isolate HS1, the DAH isolate is nearly identical to HS1, as shown by multi-locus sequencing [27] and plasmid sequences containing the silent *vmp* genes [16], [18]. The *vmp* gene in the expression site of *B. hermsii* DAH 2E7 was amplified and sequenced with primers pro and tel (Table S1) as described [16], [21], [77] and was determined to be *vlp7*. Between *vlp7* and the telomere is a *vsp26* pseudogene, which is silent in *B. hermsii* HS1 [16], [21], [77]. Transformants of low-passaged DAH 2E7 were selected with kanamycin at 100 μg/ml or with gentamicin at 20 μg/ml in mBSK-c containing 12% rabbit serum (Pel-Freez, Rogers, AR) [28], [78], incubated at 35°C and 5% CO_2_ and 3% O_2_ in a Forma Series II Water Jacket CO_2_ incubator (Thermo Fisher Scientific, Inc., Waltham, MA) with the caps loosely attached on the culture tubes. Clones were isolated by limiting dilution in liquid medium as described [28]. *Escherichia coli* TOP10 (Invitrogen, Carlsbad, CA) was used for the generation of constructs and grown in Luria broth or agar plates with kanamycin (50 μg/ml) or gentamicin (5 μg/ml).

### Genomic DNA isolation from *B. hermsii*

Genomic DNA of *B. hermsii* used for PCR was isolated from 5 ml cultures using the Wizard Genomic DNA Kit (Promega, Madison, Wi) following the instructions for Gram-negative bacteria. Genomic DNA of *B. hermsii* used for reverse-pulse-field gels or transformations was extracted from 100 ml cultures or as minipreps from 5 ml cultures as previously described [79].

### Southern blot analysis

Undigested genomic DNA was resolved by reverse-pulse-field gel electrophoresis as previously described [27] and genomic DNA (2 μg) digested with restriction enzymes was electrophoresed in a 1% agarose gel in TAE. DNA was transferred onto a MagnaGraph Nylon Membrane (Osmonics, Inc., Minnetonka, MN), and hybridized as previously described [80] with the exception that higher stringency washes were done with 0.1X SSC-0.1%SDS. Blots were prehybridized for 6 hr at 65°C and hybridized overnight at 55°C with probes to the kanamycin-resistance gene *kan* and the gentamicin-resistance gene *aacC1* (gent) [28], or at 65°C with probes to *vlp7* and *vlp36* (for detection of the plasmid lp28-1).

Hybridization probes were produced with the PCR DIG Probe Synthesis Kit (Roche Applied Science, Indianapolis, IN) following the manufacturer’s instructions. Wild-type *B. hermsii* genomic DNA was used as template for the *vlp7* and *vlp36* probes. Probes for *kan* and gent were PCR-amplified from pTABhFlgB-Kan and pTABhFlaB-Gent, respectively [28]. PCR amplification of the probes consisted of an initial denaturation at 94°C for 5 min; 35 cycles of 94°C for 30 sec, 60°C for 30 sec, 72°C for 2 min; and a final extension at 72°C for 7 min. The primers used to amplify the probes were: 15 and 16 for *vlp7*, 24 and 39 for *vlp36*, 42 and 43 for *kan*, and 40 and 41 for the gentamicin-resistance gene, *aacC1* (Table S1). The probe for *vlp7* lay in the truncated fragment of *vlp7* that remained at the expression site in the Vmp-minus mutant, Vmp^−^.

### Construction of the Vmp-minus mutant (Vmp^−^) and reconstituted (Vmp^+^R) strains

All PCR fragments larger than 1.5 kb were amplified with the Expand Long Template PCR System (Roche) while PCR products smaller than 1.5 kb were amplified with Go-Taq Flexi DNA polymerase (Promega) according to manufacturer’s instructions. PCR products and restriction-digested vectors were purified by the Qiagen PCR Purification Kit or Gel Extraction Kit (Qiagen Inc., Valencia, CA). Restriction enzymes and ligase were purchased from New England Biolabs, Inc. (Ipswich, MA). All intermediate products in plasmid constructions were sequenced using primers listed in Table S1.

Plasmid pExpKO (Fig. S3A) was constructed to delete the regulatory region of the *vmp* expression site and replace it with a kanamycin-resistance cassette by homologous recombination. Construction of pExpKO was done in three sequential steps. First, a 2.1 kb fragment upstream of the region of DNA to be deleted (Fig. 1, S3A) was amplified with primers 3 and 4 (Fig. 1, S3A, Table S1) from *B. hermsii* DAH *Spe*I-digested genomic DNA and cloned into the TOPO-XL vector (Invitrogen). Second, the kanamycin-resistance gene fused to the *B. hermsii flgB* promoter (*flgB*p*-kan*) was amplified from pTABhFlgB-Kan [28] with primers 5 and 6 (Table S1), digested with *Xho*I and *Spe*I, and ligated next to the upstream flanking region in the TOPO-XL plasmid digested with *Avr*II and *Xho*I. Finally, a 1.2 kb fragment downstream of the region of DNA to be deleted that contained the 3’ portion of *vlp7* and the 5′ portion of the pseudogene *vsp26* was amplified with primers 7 and 8 (Fig. 1, S3A, Table S1). The fragment was digested with *Sal*I and *Xba*I and ligated into the vector cut with *Xho*I and *Xba*I, yielding pExpKO (Fig. S3A). The construct was sequenced with primers 7–18 (Table S1) to confirm that no mutations in the flanking DNA had been introduced. The plasmid was transformed into wild-type *B. hermsii* DAH 2E7 (Fig. 1, WT) and the Vmp-minus mutant, Vmp^−^ (Fig. 1), was selected with kanamycin-supplemented medium (see below for further details). Mutants were confirmed by PCR with primer pairs 19/20 and 21/22, where one primer is within the *kan* gene and the other outside of the flanking DNA cloned into pExpKO.

The reconstituted strain, Vmp^+^R, was constructed by replacing the mutant lp28-1 expression plasmid with a wild-type copy of lp28-1 marked with a gentamicin-resistance cassette (Fig. 1). This required a two-step process: first construct a strain with a marked wild-type copy of the plasmid, and then use genomic DNA from this strain to transform the Vmp^−^ mutant. For the first construct a 3 kb fragment of lp28-1 was PCR-amplified from genomic *B. hermsii* DNA with primers 23 and 24 (Table S1, Fig. 1, S3B), and cloned into a TOPO-XL vector. The resulting plasmid was amplified by inverse PCR with the primers 25 and 26 (Table S1, Fig. S3B) to introduce restriction sites, and then digested with *Avr*II and *Xma*I. The gentamicin-resistance cassette (*flaB*p-*aacC1*) was amplified from pTABhFlaB-Gent [28] with primers 27 and 28 (Table S1), digested with *Xma*I and *Spe*I, and ligated into the vector and sequenced with primers 23–34 (Table S1). The resulting pLp28-1G (Fig. S3B) was transformed (see below) into the wild-type *B. hermsii* DAH 2E7 and transformants were selected with gentamicin. The marked wild-type plasmid contained the gentamicin-resistance cassette inserted between the pf50 and *tra* genes on lp28-1 and created a 71bp deletion in a non-coding region of DNA in the lp28-1 plasmid. Insertion of the *flaB*p-*aacC1* into lp28-1 was confirmed by PCR with primer pairs 35/37 and 36/38 (Table S1), where one primer was located within the gentamicin–resistance cassette and the other was located on lp28-1 outside of the region cloned into pLp28-1G. Genomic DNA was isolated from this strain and transformed into Vmp^−^ mutant strain (see below). The reconstituted mutants were selected with gentamicin, cloned by limiting dilution, and the clones were screened for loss of kanamycin resistance.

### Construction of the Vtp-minus mutant (Δ*vtp*) and reconstituted (*vtp* ^+^R) strains

The same clone (2E7) of *B. hermsii* DAH that was used to produce the Vmp^−^ mutant was also used to produce the Δ*vtp* mutant and its reconstituted strain. The methods for constructing Δ*vtp* and *vtp* ^+^R were presented previously [28].

### Transformation of *B. hermsii*

Competent *B. hermsii* cells were electroporated with 25 μg plasmid or genomic DNA as described by Battisti et al. [28] with the following changes for the Vmp^−^ and Vmp^+^R strains: The EPS buffer contained potassium phosphate (0.27 M sucrose, 15% w/v glycerol, 2.43 mM K_2_HPO_4_, 0.57 mM KH_2_PO_4_, pH 7.4) and was used at 4°C instead of RT, and the cultures were grown in a low O_2_ (3%) incubator. After 4–7 days in antibiotic selection, 1 ml of the culture was passed into 5 ml fresh medium with antibiotics, allowed to grow for 3 days, and then cloned by limiting dilution.

### Immunoblots and serology

*B. hermsii* whole cell lysates were prepared as previously described [81] and electrophoresed in a 12% Tris-Glycine gel (Life Technologies, Carlsbad, CA). Proteins were transferred onto a nitrocellulose membrane in the BioRad transblotter (Bio-Rad Laboratories, Hercules, CA) with transfer buffer (11.25 mM NaPO4-dibasic, 3.75 mM NaPO4-monobasic). Membranes were blocked overnight in TBS-T (25 mM Tris, 150 mM NaCl, 0.1% Tween-20 pH 7.4) containing 5% powdered milk. Membranes were incubated with convalescent serum samples from infected mice (diluted 1:500), mouse anti-Vtp monoclonal antibody H4825 [25] (diluted 1∶50), or mouse anti-Vlp7 monoclonal antibody H9326 [82] (diluted 1∶50) in TBS-T with 5% milk for 1 hr. Membranes were washed 3 times for 10 min each with TBS-T then incubated with HRP-rec-protein A 1∶5000 (Invitrogen) in TBS-T with 5% milk for 1 hr. Membranes were washed 3 times for 10 min with TBS-T and bound antibodies were detected with the SuperSignal West Pico Chemiluminescent Substrate (Thermo Fisher Scientific, Inc.) and visualized on film.

### QPCR quantification of spirochetes in the blood of infected mice

Adult female RML mice were from a closed colony at the Rocky Mountain Laboratories that originated from outbred Swiss-Webster mice. Severe combined immune deficiency (SCID) mice C3SnSmn.CB17-*Prkdc^scid^*/J and the background strain C3H/HeSnJ mice were purchased from The Jackson Laboratory. SCID mice were housed in isolator cages.

Mice were inoculated with 500 spirochetes by intraperitoneal (i.p.) injection or fed upon by 10 *O. hermsi* ticks infected with the wild-type, Vmp^−^ mutant, or Vmp^+^R reconstituted strains (tick-infections described below). The number of spirochetes in blood was quantified by QPCR as described by McCoy et al. [35]. Briefly, blood was collected from the mice by nicking the tip of the tail and expressing blood from the tail vein onto a slide. A 5 μl drop of blood was placed into 95 μl of SideStep Lysis and Stabilization Buffer (Agilent Technologies, Santa Clara, CA) in duplicate and stored at –80°C. When all samples were collected, the lysed cells were thawed and diluted 1∶10 in sterile distilled water and 3 μl were used as template in triplicate in QPCR using the Stratagene Brilliant II QPCR Master Mix (Agilent Technologies) with a probe and primer set to the *B. hermsii flaB* gene [35]. The number of spirochetes per ml of blood in the mice was calculated from a standard curve of a 10-fold serial dilution of a known number of spirochetes spiked into the SideStep Lysis and Stabilization buffer along with 5 μl of uninfected blood, diluted 1∶10 in sterile distilled water, and 3 μl used as template.

### Tick infections

Mice were inoculated by i.p. injection with 500 spirochetes of the wild-type or Vmp^+^R strain, or 1.5×10^8^ spirochetes of the Vmp^−^ strain and monitored daily for the first peak of infection by collecting a thick drop of blood from the tail vein. Immediately prior to tick feeding, two 5 μl drops of blood were taken from each mouse and each placed in 95 μl Lysis and Stabilization Buffer (Agilent Technologies) and frozen at –80°C for quantification of spirochetemia levels by QPCR. Approximately 75 *O. hermsi* SIS second-stage nymphs [35] were allowed to feed on each of the three infected mice to acquire the wild-type, Vmp^−^ mutant, or Vmp^+^R reconstituted strains. The Δ*vtp*, *vtp* ^+^R and wild-type spirochetes were quantified in mice prior to tick-feeding by examination of a dried blood smear stained with Giemsa and spirochetes counted by bright-field microscopy. Ticks were fed on mice that were anesthetized with pentobarbital (0.5 mg/10 g body wt) (Abbott Laboratories, North Chicago, IL) via intraperitoneal injection. The hair on the abdomen of the mouse was sheared with electric clippers and ticks were placed on the abdomen and allowed to feed to engorgement for 20 to 60 minutes. One or two ticks from each group were squashed in PBS immediately after feeding and examined by dark-field microscopy to confirm the spirochete acquisition. Engorged ticks were maintained at 85% relative humidity, 21°C and a natural photoperiod for subsequent examination and transmission experiments.

### Tick transmission experiments with the Δ*vtp* mutant

The experimental infectious cycle with ticks and mice was initiated by inoculating mice intraperitoneally with a culture suspension of wild-type, *Δvtp* mutant or the *vtp* ^+^R strain. Uninfected second nymphs and adult *O. hermsi* ticks were allowed to engorge on mice as described above. Ticks were dissected and examined for their infection and spirochete phenotype (see below) at selected time points from 7 to 448 days after feeding on infected mice (Table 1). Other ticks in the same cohorts were allowed to feed again on mice at selected times from 70 to 386 days after they had acquired spirochetes to determine the infectiousness of the three *B. hermsii* strains by tick bite (Table 3). Tick transmission experiments were performed with 5 ticks applied to the belly of 10 mice, 12 ticks on 2 mice, and 20 ticks on 4 mice. After ticks fed, the mice were examined daily for 10 days by microscopic examination of blood collected from the tail vein. When mice became spirochetemic, thin smears of blood were prepared on microscope slides for subsequent analysis to determine the serotype of the spirochetes. The 16 mice fed upon by infected ticks were kept for 3 to 4 months, at which time serum samples were collected for serological tests for anti-*B. hermsii* antibodies determined by IFA with fixed, whole cells of *B. hermsii* [83], [84].

### Indirect immunofluorescence antibody (IFA) assays

Double-labeled IFA assays were performed on thin blood smears and tick tissues to detect spirochetes and determine if the spirochetes were producing Vlp7 or Vtp. Drops of blood were collected from the tip of the tail vein of the mice as described above, thin smears were made on microscope slides, dried at RT, fixed in 100% methanol for 20 min and stored at 4°C. The midgut and salivary gland tissues from potentially infected ticks were dissected at various times after their acquisition of spirochetes. These tissues were dissected from single ticks partially immersed in PBS, rinsed and prepared on individual slides as described [23], fixed with 100% acetone and stored at 4°C until examined.

The blood and tick tissues were incubated with mouse monoclonal anti-Vtp antibody H4825 [25] or mouse monoclonal anti-Vlp7 antibody H9326 [82] undiluted for 30 min, washed in PBS for 15 min, followed by a goat anti-mouse-FITC (Kirkegaard & Perry Laboratories, Inc., Gaithersburg, MD) diluted 1∶100 for 30 min and washed again. The slides were then incubated with a rabbit hyperimmune anti-*B. hermsii* serum 2779 (unpublished) at 1∶50 for 30 min, washed for 15 min in PBS, followed by goat anti-rabbit-RITC (Kirkegaard & Perry Laboratories, Inc.) at 1∶:100 dilution. After a final wash and rinse with water, the samples were mounted with glycerol and examined with a Nikon Eclipse E800 epifluorescence microscope at 400X with two filters specific for fluorescein and rhodamine. Spirochetes were counted and scored as positive or negative for Vtp or Vlp7.

Six ticks from each cohort infected with the wild-type, Vmp^−^ mutant, or Vmp^+^R strain were dissected at 98–105 days after the ticks had fed and subsequently molted. For the Δ*vtp* experiments, 5 ticks were examined at each sampling time-point for spirochetes producing Vtp (Table 1), while just one tick per time-point was examined for spirochetes producing Vlp7 (Table 2).

## Supporting Information

Figure S1 **Southern blot analysis of digested DNA of the wild-type (WT) and two Vmp** ^−^ **mutant clones.** Two *Psi*I restriction sites flank the *flgB*p*-kan* insertion and one *Mfe*I site is located just upstream of *flgB*p-*kan* (Fig 1). Both restriction digests resulted in a fragment that was 216 bp larger in the Vmp^−^ mutants than in the wild-type (WT). Southern blots of genomic DNA digested with *Psi*I or *Mfe*I from the WT and two Vmp^−^ mutant clones were probed for *vlp7* and the kanamycin-resistance gene, *kan*. For each digest, the probe to *vlp7* hybridized with two bands in each strain: a common band corresponding to the silent *vlp7* cassette located on another linear plasmid [16] and a band corresponding to the *vlp7* at the expression site. A 0.2 kb shift in the fragment size for both restriction digests between the wild-type and the mutants indicates the mutation occurred at the telomeric expression locus of lp28-1. The probe to *kan* hybridized to the mutants on the same restriction fragment containing the *vlp7* at the expression site. If the mutation had occurred in a long expression plasmid [7], then the shift in the *Mfe*I-digested DNA would have been larger than in the *Psi*I-digested DNA. The predicted size of the *Mfe*I fragment that the *vlp7* and *kan* probes would have hybridized to in the expression site of the mutants would have been 4.2 kb (a 1.4 kb shift). Also, the *vlp7* probe to the silent cassette hybridized to the same size fragment in the wild-type and mutant strains, demonstrating that the mutation did not occur in the silent *vlp7* cassette. Therefore, the mutation and inactivation of the expression locus must have occurred near the telomere of lp28-1. Molecular size standards in kilobase pairs (kb) are on the left.
(TIF)

Figure S2 **Southern blots of undigested genomic DNA of the wild-type (WT), Vmp^−^ and Vmp^+^R strains.** Undigested genomic DNA of the three strains was resolved by reverse-pulse-field gel electrophoresis and transferred to a nylon membrane. Southern blots were probed for *vlp7*, *vlp36* (to identify the lp28-1 plasmid), the kanamycin-resistance gene (*kan*), or the gentamicin-resistance gene (*aacC1*). Agarose gel stained with Gel Red (**A**). The probe for *vlp7* hybridized to two plasmids in all strains, one containing the silent *vlp7* cassette and the other containing the *vlp7* in the expression site on lp28-1 (**B**). The *vlp36* probe hybridized to the lp28-1 plasmid in all strains (**C**). The *kan* probe hybridized only to the Vmp^−^ mutant (**D**) and the *aacC1* probe hybridized only to the Vmp^+^R strain (**E**). Molecular size standards in kilobase pairs (kb) are on the left with the Ladder Lambda DNA-MonoCut Mix (New England Biolabs).
(TIF)

Figure S3 **Plasmids used in the construction of the Vmp** ^−^ **mutant and Vmp^+^R reconstituted strains.** Plasmid pExpKO (**A**) was used in the transformation of wild-type *B. hermsii* DAH 2E7 to construct the Vmp^−^ mutant by inactivating the *vmp* expression site by homologous recombination. Plasmid pLp28-1G (**B**) was used in the transformation of wild-type *B. hermsii* DAH 2E7 to construct the wild-type lp28-1 marked with the gentamicin-resistance cassette.
(TIF)

Table S1 **Oligonucleotide primers used for PCR and sequencing.**
(DOCX)
