# Supplementary figures and images for: Inactivation of Genes for Antigenic Variation in the Relapsing Fever Spirochete Borrelia hermsii Reduces Infectivity in Mice and Transmission by Ticks
Source: PLoS Pathog. 2014 Apr 3;10(4):e1004056. doi: 10.1371/journal.ppat.1004056 (PMC3974855; doi:10.1371/journal.ppat.1004056)

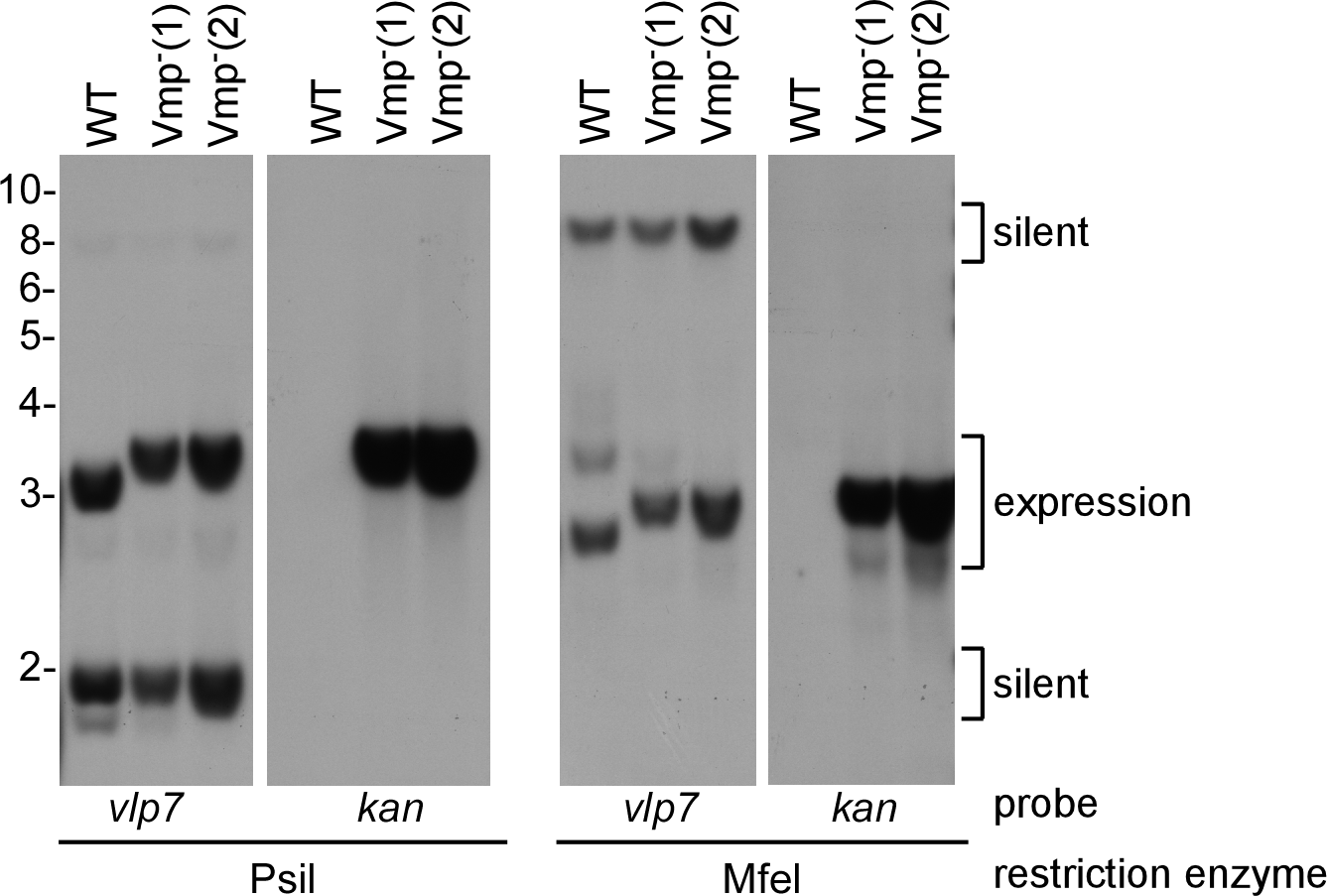

Supplement: Figure S1 — Southern blot analysis of digested DNA of the wild-type (WT) and two Vmp − mutant clones. Two PsiI restriction sites flank the flgBp-kan insertion and one MfeI site is located just upstream of flgBp-kan (Fig 1). Both restriction digests resulted in a fragment that was 216 bp larger in the Vmp− mutants than in the wild-type (WT). Southern blots of genomic DNA digested with PsiI or MfeI from the WT and two Vmp− mutant clones were probed for vlp7 and the kanamycin-resistance gene, kan. For each digest, the probe to vlp7 hybridized with two bands in each strain: a common band corresponding to the silent vlp7 cassette located on another linear plasmid [16] and a band corresponding to the vlp7 at the expression site. A 0.2 kb shift in the fragment size for both restriction digests between the wild-type and the mutants indicates the mutation occurred at the telomeric expression locus of lp28-1. The probe to kan hybridized to the mutants on the same restriction fragment containing the vlp7 at the expression site. If the mutation had occurred in a long expression plasmid [7], then the shift in the MfeI-digested DNA would have been larger than in the PsiI-digested DNA. The predicted size of the MfeI fragment that the vlp7 and kan probes would have hybridized to in the expression site of the mutants would have been 4.2 kb (a 1.4 kb shift). Also, the vlp7 probe to the silent cassette hybridized to the same size fragment in the wild-type and mutant strains, demonstrating that the mutation did not occur in the silent vlp7 cassette. Therefore, the mutation and inactivation of the expression locus must have occurred near the telomere of lp28-1. Molecular size standards in kilobase pairs (kb) are on the left. (TIF) [file ppat.1004056.s001.tif]

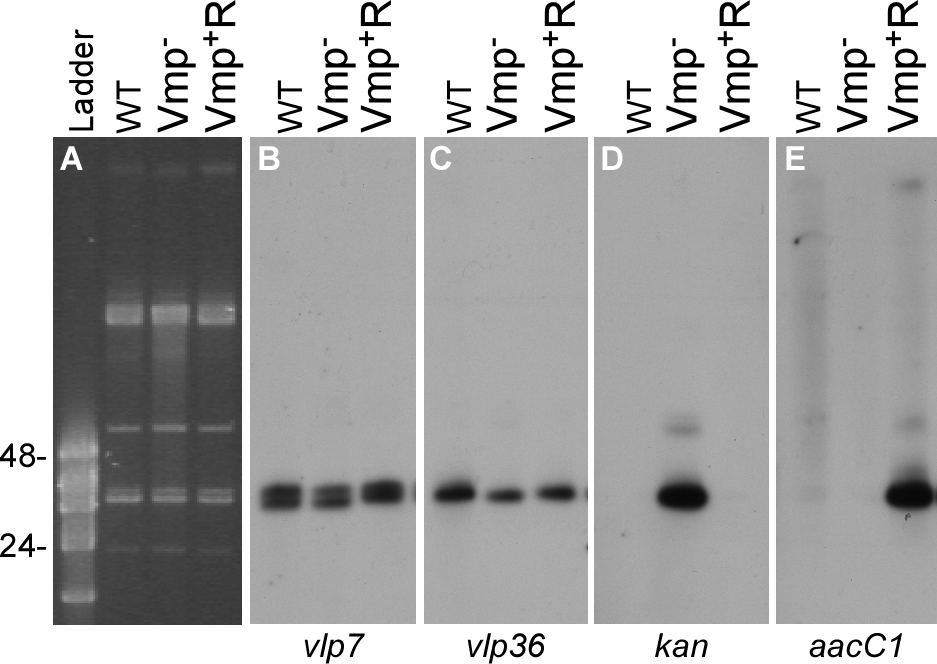

Supplement: Figure S2 — Southern blots of undigested genomic DNA of the wild-type (WT), Vmp− and Vmp+R strains. Undigested genomic DNA of the three strains was resolved by reverse-pulse-field gel electrophoresis and transferred to a nylon membrane. Southern blots were probed for vlp7, vlp36 (to identify the lp28-1 plasmid), the kanamycin-resistance gene (kan), or the gentamicin-resistance gene (aacC1). Agarose gel stained with Gel Red (A). The probe for vlp7 hybridized to two plasmids in all strains, one containing the silent vlp7 cassette and the other containing the vlp7 in the expression site on lp28-1 (B). The vlp36 probe hybridized to the lp28-1 plasmid in all strains (C). The kan probe hybridized only to the Vmp− mutant (D) and the aacC1 probe hybridized only to the Vmp+R strain (E). Molecular size standards in kilobase pairs (kb) are on the left with the Ladder Lambda DNA-MonoCut Mix (New England Biolabs). (TIF) [file ppat.1004056.s002.tif]

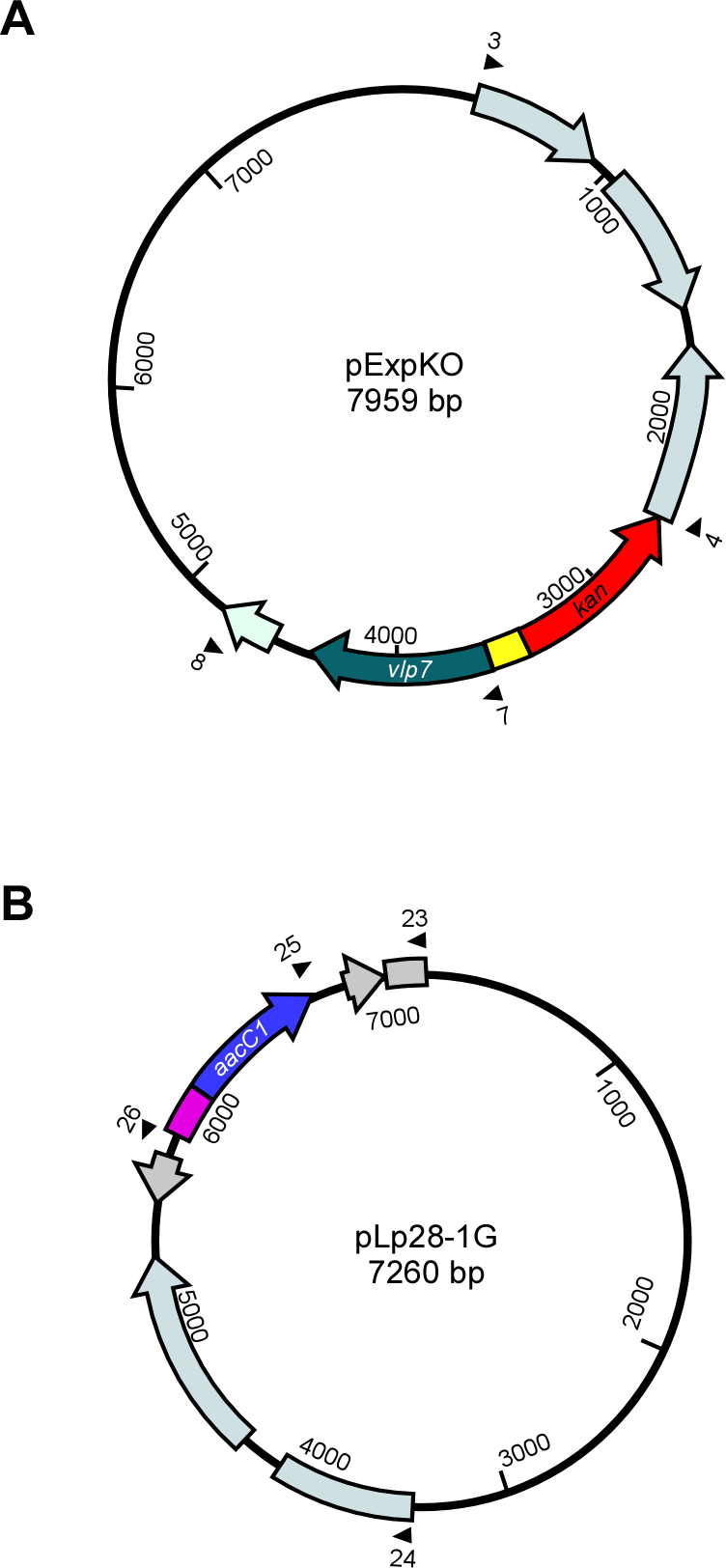

Supplement: Figure S3 — Plasmids used in the construction of the Vmp − mutant and Vmp+R reconstituted strains. Plasmid pExpKO (A) was used in the transformation of wild-type B. hermsii DAH 2E7 to construct the Vmp− mutant by inactivating the vmp expression site by homologous recombination. Plasmid pLp28-1G (B) was used in the transformation of wild-type B. hermsii DAH 2E7 to construct the wild-type lp28-1 marked with the gentamicin-resistance cassette. (TIF) [file ppat.1004056.s003.tif]
