# Supplementary material for: Inactivation of Genes for Antigenic Variation in the Relapsing Fever Spirochete Borrelia hermsii Reduces Infectivity in Mice and Transmission by Ticks
Source: PLoS Pathog. 2014 Apr 3;10(4):e1004056. doi: 10.1371/journal.ppat.1004056 (PMC3974855; doi:10.1371/journal.ppat.1004056)
Supplement: Table S1 — Oligonucleotide primers used for PCR and sequencing. (DOCX) [file ppat.1004056.s004.docx]

Table S1. Oligonucleotide primers used for PCR and sequencing.

| Primer No. | Designation | Sequence |
| --- | --- | --- |
| 1 | pro | TAAACTTTGAAAGTTGAGGTATAATGC |
| 2 | tel | TAGTACAAATCCCCTTGCCGCTTC |
| 3 | L2expF | GTGGTTCTAAAAAGGATGAGGGGG |
| 4 | KO-5’-AvrII | AAGGAT**CCTAGG**CCGTTAGTGATGCTATTGGAGATGC |
| 5 | SPM13F | CGACGTTGTAAAACGACGGC |
| 6 | SPM13R | GGAAACAGCTATGACCATGATTACG |
| 7 | KO-3'-SalI | ATGACT**GTCGAC**AAGTAGCAGTAAAAGCAGAAACAGGTG |
| 8 | KO-R-XbaI | CC**TCTAGA**TCAGCAACACTAACCACCCCTG |
| 9 | expF1 | GCGGTAGTTTTGATGCTGAAGG |
| 10 | expF2 | CAGATGGTGCTAAGGATAAAGGAG |
| 11 | expF3 | GTCAGCACCAGTTACAGCCC |
| 12 | expR1 | CTTCAGCTTCAGTCTTATTGTCACCAG |
| 13 | expR2 | CCCATCTAATTTTACCAACGTTTCC |
| 14 | expR3 | AAGTCCTGAACAGAACACCCC |
| 15 | vlp7+1 | GCAGATAATGCTACAGGAGGGG |
| 16 | vlp7-1 | CTGAAACTGGCTCTTGTGAACCTC |
| 17 | vlp7-2 | GCTCCTCCATTTGCTTGTAGTGTAG |
| 18 | vsp26+1 | TGGAACTTTGGAAGCCATAGC |
| 19 | L3expF | GTGAAGGAAGTCGTTGACAAGTTCG |
| 20 | kanout3’ | CGGATTCAGTCGTCACTCATGGTG |
| 21 | kanout5’ | GGAAGAGGCATAAATTCCGTCAGCC |
| 22 | vsp26-1 | GTTCTTCCGCTCCATTCTCCCC |
| 23 | fragI32 | GTAACTTGTAAGACTTGCTTGGGC |
| 24 | vlp36-2 | CAGTGGAATCACCATTAGCCATTGC |
| 25 | KI-5'-AvrII | AT**CCTAGG**GCATGGAAGTTATTGTCTTATCTC |
| 26 | KI-3'-XmaI | TG**CCCGGG**TTGTTTTTAGGTTGTTAATGGAC |
| 27 | gent5'-XmaI | ATTCG**CCCGGG**CCGGCAATTCCTAATCAGAAAAATGTGG |
| 28 | gent3'-SpeI | AA**ACTAGT**CTCGGCTTGAACGAATTGTTAGG |
| 29 | KI3'-F | AACAACCCCACCATCATCGG |
| 30 | KI3'-F1 | CAATCTCAGTTTCAGCTGCATTAGG |
| 31 | KI3'-F2 | TGTAATAGCGGTGGGGTTGC |
| 32 | KI3'-R | CCAAAAGTCACAAAAACATCTAAGAATC |
| 33 | KI3'-R1 | ACAAAAAAGTCTGATGTTGGGG |
| 34 | KI3'-R2 | GGTGGTGATGCTGTCAAGTTAGC |
| 35 | fragI-1680F | GAGATATAATCAAATCAGAGACAACAAGAGC |
| 36 | fragI-5691R | CGGCTGATATTACACTATGTACCCC |
| 37 | gentout | GCAAGCAGATTACGGTGACG |
| 38 | 3'BhflaBp | TCATATGTCATTTCCTCCGTG |
| 39 | vlp36-1 | CCTGCTGTTGACGATAAGAAGAAAATAGGG |
| 40 | Gent-G1 | ATCACCTCTTCCCGTATGCC |
| 41 | Gent-G2 | CAAAGTTAGGTGGCTCAAGTATGG |
| 42 | upkan | GAAAAACTCATCGAGCAT |
| 43 | lowkan | TGCCAATGATGTTACAGA |

Restriction endonuclease sites are indicated in bold. All primers are from this study except primers pro and tel [16].
